# High Expression of RAI14 in Triple-Negative Breast Cancer Participates in Immune Recruitment and Implies Poor Prognosis Through Bioinformatics Analyses

**DOI:** 10.3389/fphar.2022.809454

**Published:** 2022-04-01

**Authors:** Ranliang Cui, Ting Zhao, Changsen Bai, Ning Ji, Jialei Hua, Li Ren, Yueguo Li

**Affiliations:** Department of Clinical Laboratory, Key Laboratory of Cancer Prevention and Therapy, Tianjin’s Clinical Research Center for Cancer, National Clinical Research Center for Cancer, Tianjin Medical University Cancer Institute and Hospital, Tianjin, China

**Keywords:** retinoic acid-induced protein 14, carboxypeptidase N1, prognosis, tumor-infiltrating lymphocytes, triple-negative breast cancer

## Abstract

**Objective:** The purpose of current research is to explore the function of retinoic acid-induced protein 14 (RAI14), being a reciprocal protein of carboxypeptidase N1 (CPN1), and as a biomarker for prognosis and immunoregulatory effects in breast cancers.

**Methods:** Interacting proteins of CPN1 were characterized by co-immunoprecipitation (CO-IP) and mass spectrometry. We evaluated RAI14 expression and related clinical prognosis based on bioinformatics methods. The level of relevance between RAI14 and infiltrating immune cells biomarkers was investigated by using TIMER and certificated by immunohistochemical staining and cytology experiments.

**Results:** RAI14 is an interacting protein of CPN1. Higher RAI14 expression in TNBC was significantly correlated with poor prognosis in TNBC, especially (RFS: HR = 1.32, *p* = 0.015; DFS: HR = 1.18, *p* = 0.035). The estrogen receptor (ER), P53 status, and histological types and triple-negative status were observed and correlated with RAI14 expression. Moreover, the level of RAI14 was positive in relation with the expression of CD163 (M2 macrophages marker, r = 0.393, *p* = 1.89e-06) and PD-1 (T-cell exhaustion marker, r = 0.626, *p* = 4.82e-03), indicating RAI14 levels were mainly related to M2 macrophages and T-cell exhaustion infiltration in TNBC. Furthermore, CPN1 overexpression was accompanied by RAI14 and PD-L1 upregulation, and a correlation was found among them.

**Conclusions:** RAI14 is a potential downstream molecule of CPN1, which may be a potential prognostic biomarker and identification of an immunosuppressive tumor microenvironment in TNBC.

## Introduction

TNBC is an advanced metastatic pattern of mammary tumor that is related to poor prognosis, making this subtype of breast cancer nearly fatal (Duan et al., 2016; Wang et al., 2020a). Due to lack of specific therapeutic targets and resistance to first-line chemotherapy, immunotherapeutic intervention is a selectable approach which has been shown to be more sensitive in TNBC in contrast to other molecular types of breast cancer (Stamm et al., 2019).

Immunity checksites are a group of inhibitory molecules that can cause immune escape serve as available versions of immunotherapy (Niknam et al., 2018). PD-1 mediates immune tolerance of peripheral T cells (Bour-Jordan et al., 2011). Recent studies in a variety of epithelial cancers have shown that PDCD1 is presented in infiltrating immune cells and PD-L1 is expressed on cancer cells (Keir et al., 2008; Muenst et al., 2014). The PD-1/PD-L1 pathway is a major contributor to cancer progression by suppressing anti-cancer immune responses (Billon et al., 2019), which implies that PD1/PDL1 is an important factor that causes evasion of immunity (Dong et al., 2002; Iwai et al., 2002). In a study analyzing samples, PD-L1 was reported to be found in 34% of mammary tumors (Ghebeh et al., 2006). Anti-PD-L1 as a combination drug is mostly used in the treatment of progressive TNBC (Zhang et al., 2019; Björk Gunnarsdottir et al., 2020). Hence, immunity checksites may help predict the prognosis of cancer inpatients (de Andrea et al., 2018).

In addition, growing evidence indicated that innate immunocytes could mediate angiogenesis, proliferation, survival, and invasion *via* secretion of cytokines, thereby affecting the prognosis and effectiveness of chemotherapy and immunotherapy (Waniczek et al., 2017; Zhang et al., 2018; Ham et al., 2019). Nevertheless, less detailed investigation about immunocytes with key molecules on TNBC prognosis and immunotherapy was carried out.

Previously, we have found that the overexpression of carboxypeptidase N1 (CPN1) facilitated metastasis in breast cancer cells (Cui et al., 2020). Furthermore, preliminary serologic experiments demonstrated that CPN1 is highly expressed in the serum of breast cancer patients, which is significantly related to metastasis of TNBC (Cui et al., 2020). In the present study, we have verified that CPN1 and retinoic acid-induced protein 14 (RAI14) are interacting proteins by using CO-IP and bioinformatics. Moreover, it has been reported that RAI14 was highly expressed and may enhance translocation of esophageal tumor cells (Wang et al., 2020b)^.^ These studies indicate that RAI14 plays a significant function in cancer progression, indicating the oncogenic characteristic (Xiao et al., 2020). Nevertheless, the underlying functions and mechanisms of RAI14 regarding cancer progression and tumor immunology remain uncertain.

In the current research, we generally investigated high-RAI14 which is associated with worse survival of breast cancer with the assistance of some databases based on bioinformatics methods. Moreover, the correlation between RAI14 levels and various pathological parameters was determined. Subsequently, we examined the relationship between differential expression of RAI14 and the prognosis of mammographic variations. Furthermore, we analyzed the correlation between RAI14 expression and immune infiltration gene markers using the TIMER database. Ultimately, validated experiments were made by cytology and immunohistochemistry. Our results indicated that RAI14 is the identification of prognostic biomarkers in relation to immune infiltration in TNBC.

## Materials and Methods

### Bioinformatics Database Analysis

The study showed the relevance of RAI14 with the tumor-infiltrating cells using the TIMER2.0 online tool (https://cistrome.shinyapps.io/timer/) (Li et al., 2020). In the correlation module, we have detected the correlation of RAI14 expression in different breast cancer molecular states with immune cell markers to determine the presence of any corresponding immune cell infiltration. The correlation analysis of immunological infiltration based on the Timer database. X-axis represents the expression of immune cell gene markers, and Y-axis represents RAI14.Log2TPM indicates gene expression.

The differential expression analysis of RAI14 was determined using the single gene analysis module of GEPIA (http://gepia.cancer-pku.cn/) (Tang et al., 2017). The immunohistochemical map of RAI14 was determined using HPA (https://www.proteinatlas.org/) genome-wide analysis tools (Colwill et al., 2011). The levels of RAI14 in different clinicopathological parameters (age, molecular typing, and pathological stage) of clinical breast cancers were examined using the UALCAN (http://ualcan.path.uab.edu/) (Zhang et al., 2020a). RAI14 levels in different subtypes of breast cancer were determined using Breast Cancer Gene-Expression Miner v4.7 (http://bcgenex.ico.unicancer.fr/) (Alsaleem et al., 2020). The Kaplan–Meier plotter database (http://kmplot.com/analysis/) is used to discover and validate survival biomarkers based on meta-analysis (Zhang et al., 2020b). We selected the K–M plotter at this site to analyze RAI14 expression for survival analysis.

### Immunohistochemical Staining

RAI14 and CPN1 expressions of 90 breast cancer tissue samples were determined using the tissue microarray technique and immunohistochemical staining (Cui et al., 2021). The primary antibodies for CPN1 (Wuhan Sanying Biotechnology, Hubei, China. 13385-1-AP, 4.5 μg/ml) and RAI14 (Beijing BoaoSen Biotechnology, Beijing, China, bs-19728R, 5 μg/ml), secondary antibodies (Beijing Zhongshan Jinqiao, Beijing, China), DAB color development, and modified hematoxylin staining, sealing the sections with neutral glue, were used for the experiments. The sections were identified using the following criteria. The expression of CPN1 and RAI14 in five random regions was recorded by light microscopy (20X microscopy). Positive intensity: colorless, yellow, yellow–brown, and tan, representing fractions with 0, 1, 2, and 3, respectively. The proportion of positive cells: no staining cells, 0–10%, 10–30%, 31–60%, and >60%, representing scores of 0, 1, 2, and 3. Final scoring was determined by multiplying the intensity and staining area. An IHC score ≤4 was considered to indicate a low expression of CPN1, while scores from 5 to 9 were considered to support a high level.

### Cell Culture

Breast cancer cell lines MDA-MB-231 and T47D (ATCC) were grown in RPMIA1640 (CORNING) containing 10% FBS, and breast cancer cell line MCF-7 and human renal epithelial cell line 293T (ATCC) were cultured in Dulbecco’s modified Eagle medium (DMEM, CORNING) supplemented with 10% FBS (SERATECH). All the cells were cultured at 37°C and 5% CO2 in a cell culture incubator.

### Plasmids and Transfection

The CPN1 overexpression plasmid was pCMV3-SP-Flag-CPN1 and the control was p-CMV3-SP-Flag purchased from Sino Biological Inc. The CPN1 expression plasmid was transfected into cells by lipofectamine 8000. The cell transfection experiments were performed exactly according to the instructions. The principle is that the positively charged liposome surface, which is able to interact with negatively charged DNA molecules, binds to the negatively charged phospholipid bilayer to form a DNA-lipid complex. Endocytosis of the cell and lysosomal phagocytosis allow DNA to enter the cell.

### Immunoprecipitation

The Co-IP Kit (Thermo Fisher Scientific, Waltham, MA, United States) was designed for the detection of interactions among components of the target protein complex. In total, 293T cells are used for experiments and the following steps are performed according to the kit instructions. The antigen–antibody complexes were finally eluted. A portion of the protein solution is sent for mass spectrometry (UW BGI) to detect the interacting protein with CPN1. A portion was subjected to SDS-PAGE.

### Western Blot

The relative expression levels of CPN1 and RAI14 were determined using the Western blot assay as we described previously (Yang et al., 2017). In brief, the following primary antibodies were used: CPN1 antibody (Abcam, Cambridge, MA, United States, ab232802), RAI14 antibody (Proteintech, Rosemont, IL, United States, 17507-1-AP), anti-Tubulin (Abcam, 5335s), and horseradish peroxidase-labeled secondary antibody (Cell Signaling Technology, Danvers, MA, United States). All the trials were duplicated three times. Grayscale values were measured for strip intensity using ImageJ software.

### Statistical Analysis

The Kaplan–Meier plotter was employed to resolve the difference of the survival curve with RAI14. The log-rank test results for HR and *P* or Cox *p* values are shown using the bioinformatics database. The correlation analysis among gene levels using a rank correlation coefficient. *p* < 0.05 shows statistical significance (Peng et al., 2021). GraphPad Prism version 8.0.1 and Adobe Illustrator 2020 were used for correlation analysis and graphical processing.

## Results

### RAI14 Interacts With CPN1

**FIGURE 1:**
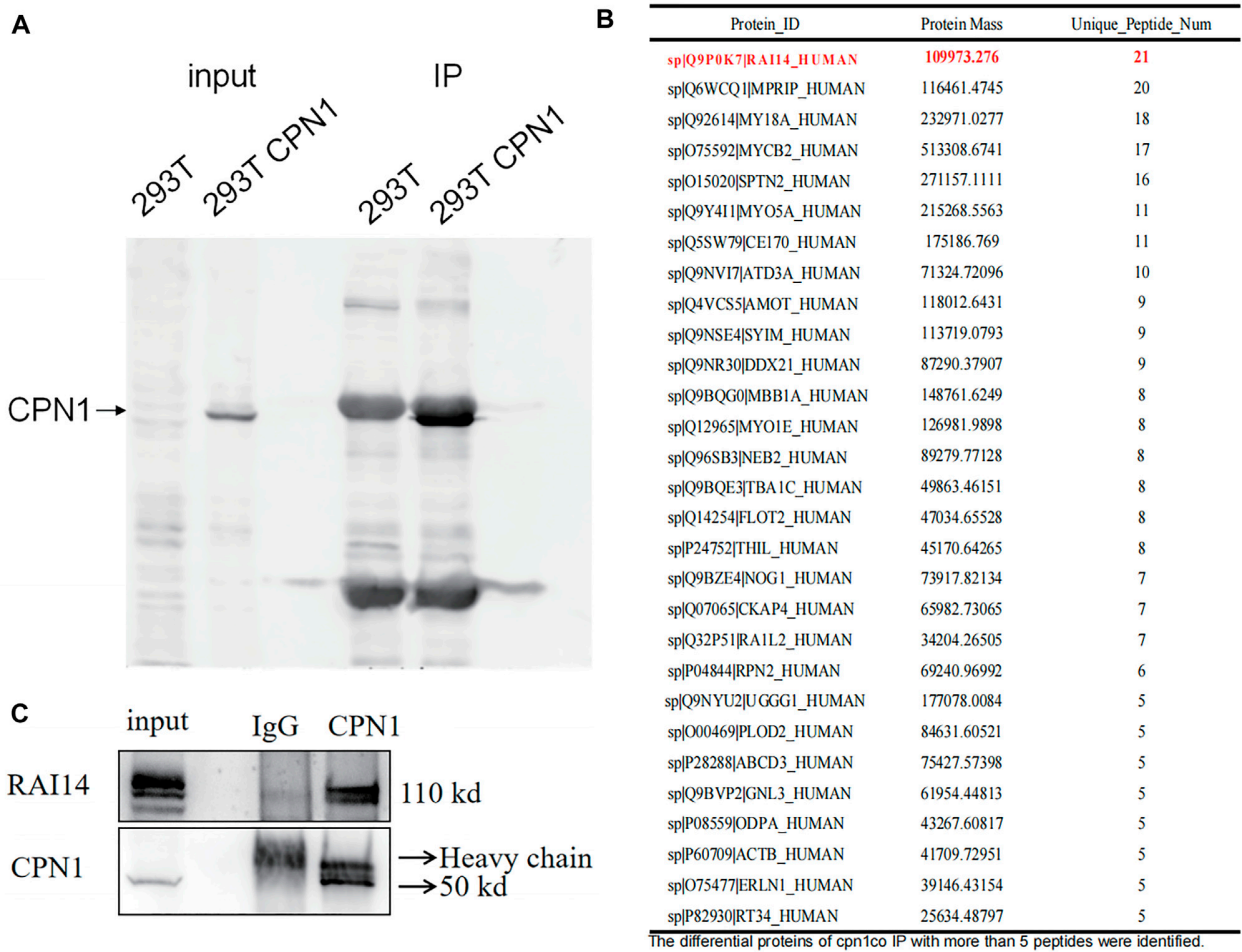
Correlation between CPN1 and RAI14. **(A)** Co-immunoprecipitation (Co-IP) in 293T cells with CPN1 overexpression. (B) A total of 29 differential proteins with more than identified five peptides in immunoprecipitation. **(C)** Validation of CPN1 interacting with RAI14.

Non-specific homologous antibody IP experiments were first performed, and the IgG group works as a negative control panel. According to these outcomes from the input group in gel electrophoresis examination, a CPN1-high group was observed in 293T cells than that in the empty plasmid group. In the IP group, the protein complexes were eluted with CPN1 antibody, and thus indicated higher amounts of interacting proteins in the CPN1 overexpression group relative to the corresponding control group (Figure 1A). Subsequently, 29 different proteins with more than five peptides were identified in immunoprecipitation (Figure 1B). Among the proteins with the discrepancy, we selected the protein that identified the most peptides as the most plausible one, in which the top ranked protein was RAI14. The immunoprecipitation results in Figure 1C demonstrated that RAI14 serves as an interacting protein with CPN1.

### RAI14 Is Highly Expressed in Breast Cancer Especially TNBC

**FIGURE 2:**
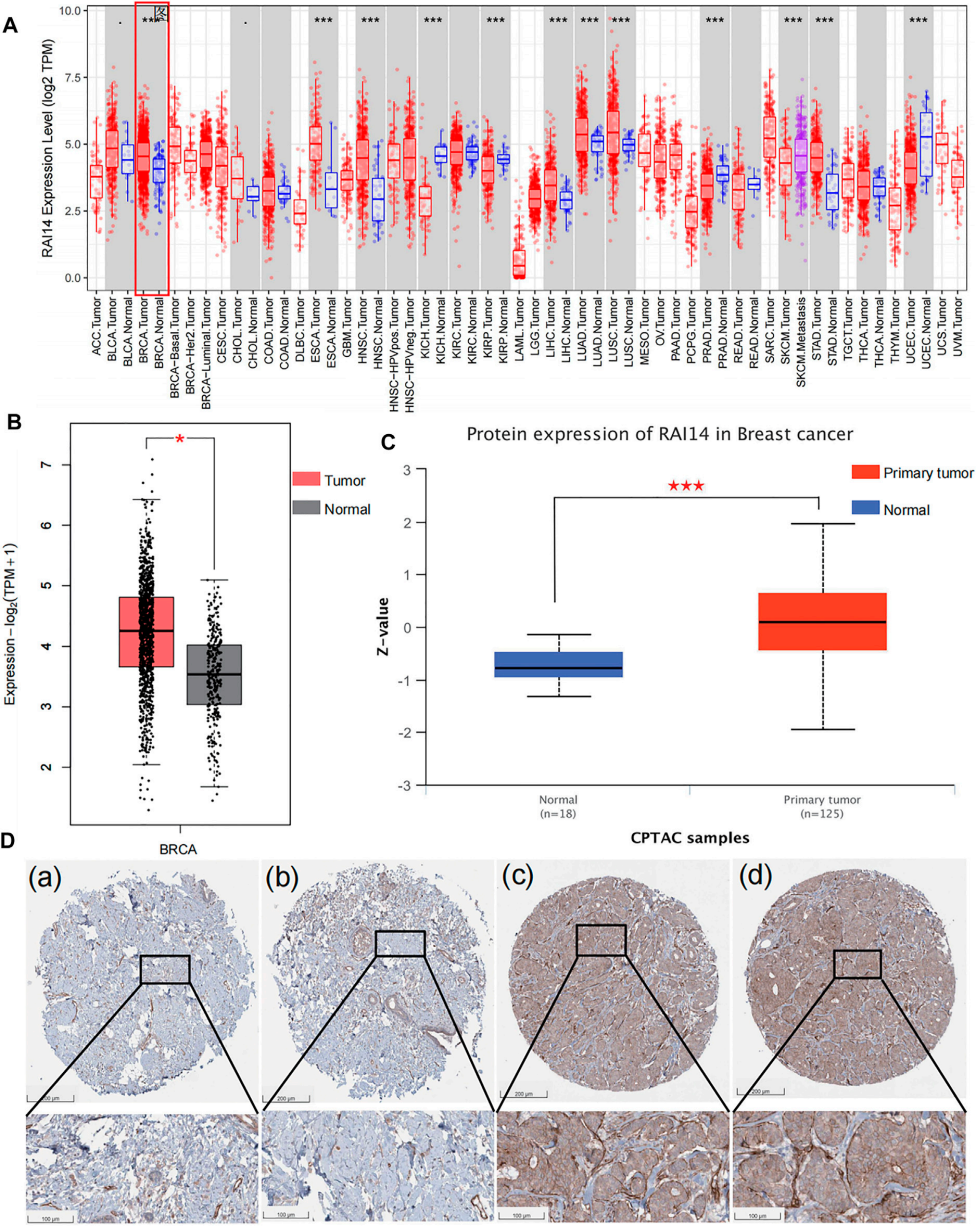
High expression of RAI14 in breast cancer.**(A)** Expression levels of human RAI14 in different tumor types from TCGA database.**(B)** The mRNA expression of RAI14 in breast cancer and normal tissues from GEPIA. **(C)**The protein expression of RAI14 in breast cancer and normal tissues from UALCAN database. **(D)** High expression of RAI14 in human breast cancer from Human Protein Atlas (HPA) database. *p < 0.05, ***p < 0.001.

According to the RNA-seq data in Tumor Immune Estimation Resource (TIMER), RAI14 levels were significantly different in cancer and paracancerous cells such as breast cancer cells (Figure 2A). RAI14 is significantly upregulated in breast cancer (BRCA) than that in paraneoplastic tissues. Furthermore, based on the information in GEPIA and ULCAN databases, both protein and mRNA expression of RAI14 is stimulated in breast cancer, which is compatible with the information from the TCGA database (Figures 2B,C). Subsequently, the expression of RAI14 was examined from the tissue by HPA. As presented in Figure 2D, a higher expression level of RAI14 was found in breast cancer tissues (Figures 2Dc,d) than that in normal tissues (Figure 2Da,b). Overall, the aforementioned results suggested that RAI14 expression level was higher in paracancer at the protein, mRNA, and tissues levels.

**FIGURE 3:**
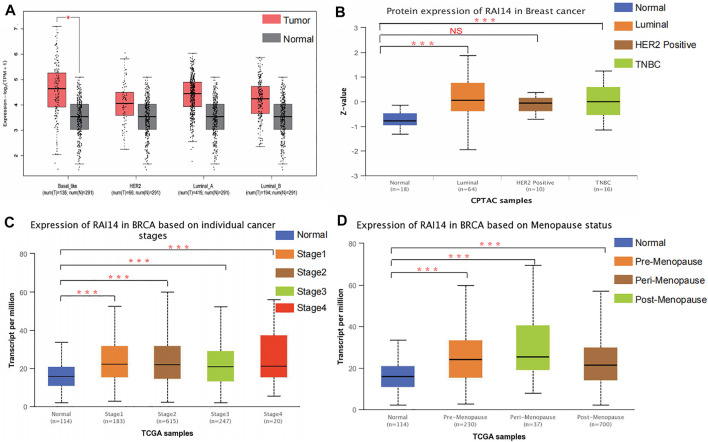
Correlation analysis of RAI14 expression levels with pathological type parameters of breast cancer. **(A)** The mRNA levels of RAI14 in mammary carcinoma with different molecular types. **(B)** Protein levels of RAI14 in mammary carcinoma with different molecular types. **(C)** Expression of RAI14 in different mammary carcinoma stages. **(D)** RAI14 levels in different menopausal states. *p < 0.05, ***p < 0.001.

The relationship between RAI14 and clinicopathological indicators was investigated at protein and mRNA levels to further determine whether RAI14 may be acted as a prospective biomarker for mammary cancer. The RNA-seq data with standard processing were analyzed by using GEPIA (Jin et al., 2020), and the differential RAI14 levels in mammary tumor with various molecular types and paraneoplasia were obtained (Figure 3A). RAI14 was highly expressed in basal-like breast carcinoma [molecules expressed as ER (-)/PR (-)/HER-2 (-), equivalent to triple-negative breast cancer] than that in paracancerous tissue. At the protein level, RAI14 expression was also dramatically upregulated in TNBC relative to the normal group (Figure 3B). In addition, the RAI14 levels in different stages of breast cancer showed high expression than normal (Figure 3C). Interestingly, we discovered that the expression of RAI14 was higher in the perimenopausal stage than that in postmenopausal stages (Figure 3D).

### Relationship Between RAI14 Expression and Clinical Pathological Parameters of Patients With Breast Cancer

**FIGURE 4:**
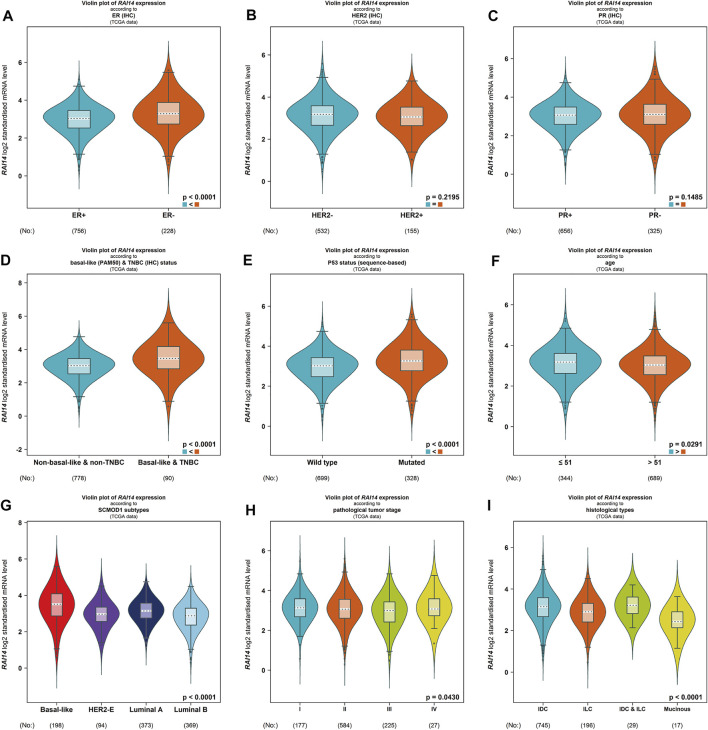
Differential expression of RAI14 in mammary carcinoma specimens with different clinicopathological parameters from BC-GenExMiner v4.4 data analysis. **(A–C)** Receptor status (ER + vs. ER-, PR + vs. PR-, HER2+ vs. HER2-).**(D)** The differential expression of RAI14 in the TNBC and non-TNBC. **(E–I)** The differential expression of **(E)** wild-type and mutant, **(F)** age (≤51 and >51 years), **(G)** molecular types, **(H)** clinical tumor stage (I, II, III, IV), and **(I)** clinicopathological type (IDC, ILC).

The relationship between RAI14 and clinicopathological parameters was further investigated using Breast Cancer Gene-Expression Miner v4.7 website (Table 1). RAI14 expression was higher in ER-breast cancer (*p* < 0.0001) (Figure 4A), while PR and HER2 status may not affect the RAI14 expression level (Figures 4B,C). The database also reveals that the expression of RAI14 was higher in TNBC than non-TNBC (*p* < 0.0001) (Figure 4D). In addition, P53 mutants, relative to the wild type, has a higher expression level of RAI14 (Figure 4E). In various typologies of breast cancer, RAI14 levels were highly expressive in TNBC than other types (*p* < 0.0001) (Figure 4G). The expression of RAI14 is significantly different for invasive lobular carcinoma and invasive ductal carcinoma (*p* < 0.0001) (Figure 4I). However, there was no difference in RAI14 expression among different age or stage (Figures 4F,H).

**TABLE 1:** Relationship between mRNA expression of RAI14 and clinicopathological parameters of breast cancer.

Variable	N	RAI14
		*p*-value
Estrogen receptor status (ER) (IHC)		
-	756	<0.0001
+	228	—
HER2 receptor status (HER2) (IHC)		
-	532	0.2195
+	155	—
Progesterone receptor status (PR) (IHC)		
-	656	<0.0001
+	325	—
Basal-like (PAM50) and TNBC (INC) status triple-negative status		
Non-basal-like and non-TNBC	778	<0.0001
Basal-like and TNBC	90	—
P53 status (sequence-based)		
Wide type	699	<0.0001
Mutated	328	—
Age (years)		
≤51	344	0.0291
>51	689	—
SCMOD1 subtypes		
Basal-like	198	<0.0001
HER2	94	<0.0001
Luminal A	373	<0.0001
Luminal B	369	<0.0001
Pathological tumor stage		
Ⅰ	177	0.0430
Ⅱ	584	—
Ⅲ	225	—
Ⅳ	27	—
Histological types		
IDC	745	<0.0001
ILC	196	—
IDC and ILC	29	—
Mucinous	17	—

### RAI14 Serves as an Important Factor in the Prognosis of TNBC

**FIGURE 5:**
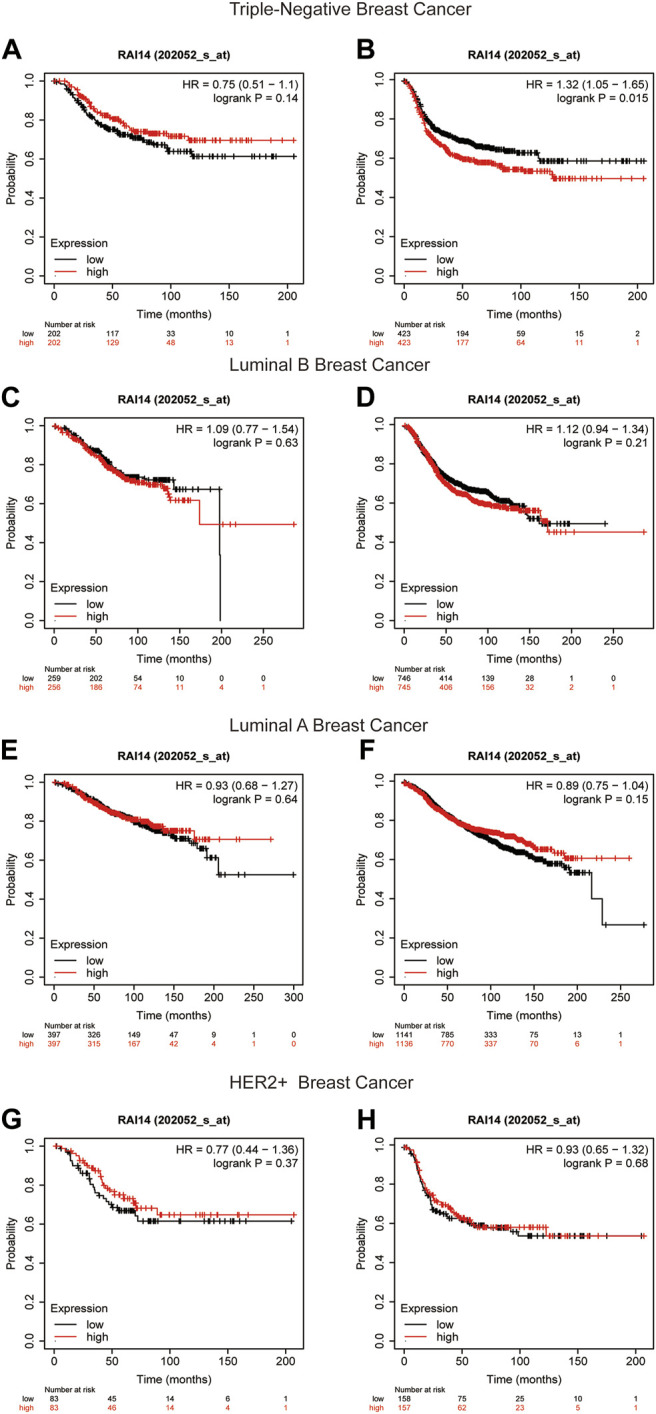
Kaplan–Meier survival curves of RAI14 in different breast cancer molecular subtypes. **(A, B)** Survival curves for overall survival (OS) and recurrence-free survival (RFS) in triple-negative breast cancer (n = 1494). **(C, D)** Survival curves for OS and RFS in luminal B breast cancer (n = 2015). **(E, F)** Survival curves for OS and RFS in luminal A breast cancer (n = 3511). **(G, H)** Survival curves for OS and RFS in HER2+ breast cancer for OS and RFS (n = 515).

The Kaplan–Meier plotter was used to compare the expression of RAI14 in different breast cancer subtypes. The database showed that the patients with high-RAI14 level had a poorer OS, RFS, and DFS (*p* < 0.05) than those with a low CPN1 expression (Figures 5A,B, Supplementary Figure S2), implying that the expression level of RAI14 may affect the survival of TNBC. However, there was no relationship between the RAI14 level and cancer prognosis in the other breast cancer subtypes (Figures 5C–H). Therefore, RAI14 has the potential to be a predictor of TNBC patient prognosis.

### Correlation of RAI14 With Infiltrative Immune Cell Markers in TNBC

**FIGURE 6:**
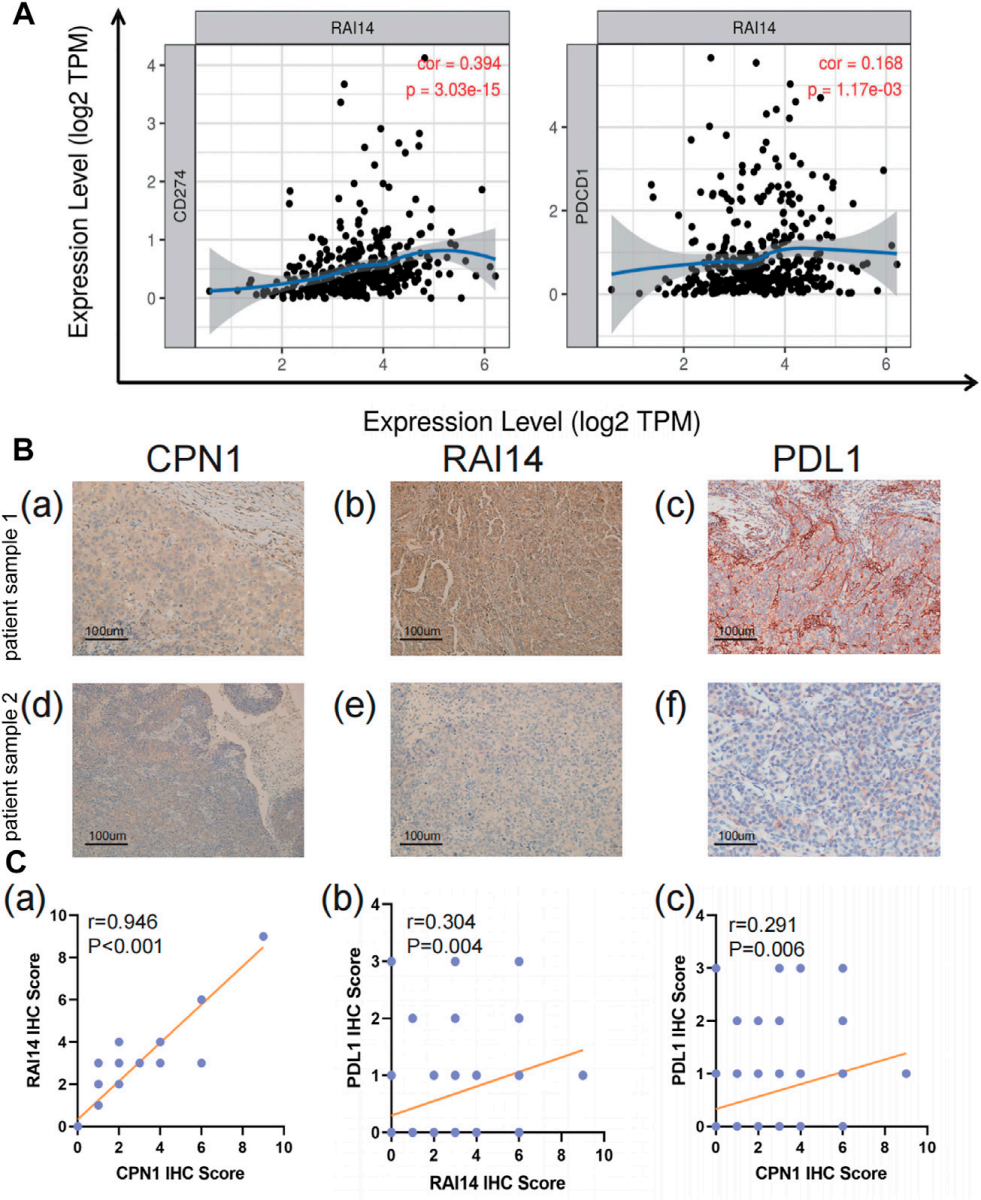
Correlation of RAI14 levels with immune infiltration in TNBC. **(A)** The expression level of RAI14 was positively correlated with programmed cell death-ligand 1 (CD274) and programmed cell death protein 1(PDCD1). X-axis represents the expression level of immune cell gene markers, and Y-axis represents RAI14. Log2TPM that indicates gene expression level. **(B)** The expression of immunohistochemical (IHC)staining of CPN1, RAI14, and PDL1 in patients with TNBC tissues. **(C)** Correlation among IHC scores of CPN1, RAI14, and PDL1.

The relationship of RAI14 expression on tumor-infiltrating immune cell markers in the different molecular subtypes was analyzed using the TIMER database (Table 2). RAI14 showed a positive correlation with the expression of PD-L1 in TNBC (Figure 6A). Furthermore, immunohistochemical (IHC) staining was employed in analyzing the relevance between RAI14 levels and immune biomarkers. The results showed that RAI14 was positively associated with PDCD1 in TNBC (Figure 6B). To further investigate the potential correlation of RAI14 and CPN1, IHC staining on tissue microarrays from 90 breast cancers was indicated. Similarly, the RAI14 and CPN1 levels were positively correlated among IHC scores of CPN1, RAI14, and PD-L1 in staining (Figure 6C).

**TABLE 2:** Correlation analysis between RAI14 and related genes and markers of immune cells in breast cancer.

Description	Gene markers	BRCA-luminal (n = 611)				BRCA-HER2+(n = 67)				BRCA-BASAL			
		None		Purity		None		Purity		None	Purity	None	Purity
		Cor	*p*-value	Cor	*p*-value	Cor	*p*-value	Cor	*p*-value	Cor	*p*-value	Cor	*p*-value
B cell	CD19	−2.19E-02	5.88E-01	−1.16E-01	6.71E-03	−2.00E-01	1.04E-01	−3.33E-01	1.06E-02	1.26E-01	1.38E-01	−1.48E-02	8.69E-01
	CD79A	−2.17E-02	5.92E-01	−1.34E-01	1.76E-03	−1.55E-01	2.11E-01	−2.80E-01	3.34E-02	1.26E-01	1.39E-01	−1.15E-02	8.98E-01
T cell (general)	CD3D	3.80E-02	3.46E-01	−7.91E-02	6.50E-02	−7.43E-02	5.49E-01	−2.14E-01	1.07E-01	7.38E-02	3.86E-01	−8.48E-02	3.41E-01
	CD3E	5.27E-02	1.91E-01	−6.85E-02	1.10E-01	−1.20E-01	3.35E-01	−2.61E-01	4.77E-02	1.05E-01	2.18E-01	−4.35E-02	6.26E-01
	CD2	8.17E-02	4.28E-02	−2.88E-02	5.03E-01	−3.92E-02	7.52E-01	−2.06E-01	1.20E-01	1.51E-01	7.47E-02	2.57E-02	7.73E-01
CD8+ T cell	CD8A	4.16E-02	3.02E-01	−6.19E-02	1.49E-01	−5.32E-02	6.69E-01	−1.94E-01	1.45E-01	9.60E-02	2.59E-01	−1.67E-02	8.52E-01
	CD8B	2.72E-02	5.00E-01	−7.97E-02	6.31E-02	−6.54E-02	5.98E-01	−1.91E-01	1.51E-01	7.95E-02	3.50E-01	5.35E-02	5.49E-01
Monocyte	CD86	2.84E-01	8.87E-13	2.30E-01	5.44E-08	3.66E-01	2.47E-03	3.26E-01	1.24E-02	3.24E-01	1.05E-04	2.55E-01	3.73E-03
TAM	CCL2	2.31E-01	7.79E-09	1.68E-01	7.92E-05	3.25E-01	7.57E-03	3.19E-01	1.46E-02	3.32E-01	6.84E-05	2.65E-01	2.52E-03
	CD68	2.81E-01	1.44E-12	2.38E-01	1.81E-08	3.80E-01	1.65E-03	3.80E-01	3.24E-03	3.52E-01	2.29E-05	3.08E-01	4.13E-04
	IL10	2.49E-01	3.45E-10	1.95E-01	4.29E-06	2.50E-01	4.14E-02	1.80E-01	1.75E-01	3.15E-01	1.59E-04	2.33E-01	8.09E-03
M1 macrophage	NOS2	2.83E-01	8.61E-13	2.72E-01	1.06E-10	2.39E-01	5.14E-02	2.43E-01	6.62E-02	1.52E-01	7.32E-02	1.39E-01	1.17E-01
	IRF5	1.60E-01	6.92E-05	1.54E-01	2.96E-04	1.18E-01	3.41E-01	8.73E-02	5.15E-01	2.76E-01	9.84E-04	1.98E-01	2.48E-02
M2 macrophage	CD163	1.85E-01	4.16E-06	1.25E-01	3.46E-03	3.33E-01	6.08E-03	3.21E-01	1.39E-02	3.93E-01	1.89E-06	3.47E-01	6.10E-05
	VSIG4	2.26E-01	1.45E-08	1.72E-01	5.41E-05	4.16E-01	5.09E-04	4.52E-01	3.70E-04	3.45E-01	3.37E-05	2.97E-01	6.50E-04
												
	MS4A4A	1.81E-01	6.04E-06	1.05E-01	1.39E-02	3.00E-01	1.39E-02	2.87E-01	2.89E-02	2.86E-01	6.39E-04	2.45E-01	5.37E-03
Neutrophils	CEACAM8	−4.26E-02	2.92E-01	−2.76E-02	5.20E-01	1.03E-01	4.07E-01	1.24E-01	3.54E-01	8.63E-02	3.11E-01	1.05E-01	2.40E-01
	ITGAM	3.12E-01	2.96E-15	2.47E-01	5.45E-09	2.74E-01	2.51E-02	2.47E-01	6.12E-02	3.49E-01	2.68E-05	2.92E-01	8.18E-04
	CCR7	−1.99E-02	6.22E-01	−1.39E-01	1.16E-03	−2.12E-01	8.47E-02	−4.04E-01	1.65E-03	1.16E-01	1.72E-01	−3.08E-02	7.30E-01
Natural killer cell	KIR2DL1	6.83E-03	8.66E-01	−4.16E-02	3.32E-01	−1.98E-03	9.87E-01	−3.19E-02	8.12E-01	9.12E-02	2.84E-01	2.95E-02	7.41E-01
	KIR2DL3	1.71E-02	6.71E-01	−3.87E-02	3.67E-01	−1.51E-01	2.22E-01	−1.54E-01	2.50E-01	6.81E-02	4.24E-01	−2.09E-02	8.15E-01
	KIR3DL1	4.99E-02	2.16E-01	−3.64E-02	3.96E-01	1.06E-01	3.93E-01	7.09E-02	5.97E-01	6.46E-02	4.49E-01	6.46E-02	4.49E-01
	KIR3DL2	−5.43E-02	1.78E-01	−1.23E-01	4.18E-03	−1.11E-01	3.71E-01	−2.42E-01	6.69E-02	4.79E-02	5.74E-01	−4.16E-02	6.41E-01
	KIR3DL3	2.94E-03	9.42E-01	−4.23E-02	3.24E-01	−4.73E-02	7.04E-01	−8.19E-02	5.41E-01	−2.96E-02	7.28E-01	−1.09E-01	2.22E-01
	KIR2DS4	1.62E-02	6.87E-01	−2.82E-02	5.12E-01	−3.05E-02	8.07E-01	−2.22E-02	8.69E-01	5.69E-02	5.04E-01	−1.15E-02	8.97E-01
Dendritic cell	HLA-DPB1	1.79E-01	7.70E-06	8.49E-02	4.76E-02	4.99E-02	6.88E-01	−1.05E-01	4.33E-01	1.38E-01	1.04E-01	1.14E-02	8.98E-01
	HLA-DQB1	1.76E-01	1.20E-05	9.31E-02	2.98E-02	1.58E-01	2.01E-01	7.07E-02	5.98E-01	9.50E-02	2.64E-01	−3.67E-02	6.81E-01
	HLA-DRA	2.54E-01	1.95E-10	1.69E-01	7.24E-05	1.76E-01	1.55E-01	6.21E-02	6.43E-01	1.97E-01	1.99E-02	9.11E-02	3.07E-01
	HLA-DPA1	2.31E-01	7.73E-09	1.49E-01	4.65E-04	1.61E-01	1.94E-01	6.09E-02	6.50E-01	1.81E-01	3.20E-02	7.88E-02	3.77E-01
	CD1C	1.11E-01	5.95E-03	3.18E-02	4.59E-01	−6.92E-02	5.78E-01	-2.19E-01	9.93E-02	1.67E-01	4.81E-02	5.55E-02	5.34E-01
	ITGAX	3.21E-01	3.43E-16	2.81E-01	2.51E-11	2.81E-01	2.17E-02	2.79E-01	3.41E-02	3.27E-01	8.62E-05	2.74E-01	1.72E-03
Th1	TBX21	1.73E-02	6.68E-01	−1.01E-01	1.83E-02	−7.68E-02	5.36E-01	−2.27E-01	8.72E-02	1.39E-01	1.03E-01	2.47E-02	7.82E-01
	STAT4	1.99E-01	6.77E-07	1.01E-01	1.82E-02	−3.16E-02	7.99E-01	−1.57E-01	2.39E-01	1.88E-01	2.61E-02	7.39E-02	4.07E-01
	STAT1	1.27E-01	1.56E−03	8.14E-02	5.75E-02	1.59E-01	1.98E-01	5.47E-02	6.84E-01	2.98E-01	3.72E-04	2.17E-01	1.41E-02
	IFNG	3.96E-02	3.26E-01	−3.58E-02	4.05E-01	5.09E-02	6.83E-01	−6.81E-02	6.11E-01	8.58E-02	3.13E-01	−1.55E-02	8.62E-01
	TNF	1.35E-01	7.67E-04	1.16E-01	6.90E-03	1.37E-01	2.68E-01	8.11E-02	5.45E-01	2.91E-01	5.18E-04	2.18E-01	1.34E-02
Th2	GATA3	−1.08E-01	7.06E-03	−2.97E-02	4.89E-01	−9.29E-02	4.54E-01	−2.16E-01	1.04E-01	−4.79E-03	9.55E-01	−3.12E-02	7.27E-01
	STAT6	1.67E-01	3.14E-05	1.85E-01	1.42E-05	1.20E-01	3.34E-01	3.21E-02	8.11E-01	2.14E-01	1.14E-02	1.26E-01	1.57E-01
	STAT5A	2.77E-01	2.37E-12	2.56E-01	1.43E-09	1.80E-01	1.45E-01	7.88E-02	5.57E-01	1.08E-01	2.04E-01	6.33E-02	4.78E-01
	IL13	9.09E-02	2.41E-02	8.20E-02	5.58E-02	4.56E-02	7.14E-01	5.91E-02	6.60E-01	3.90E-02	6.48E-01	−3.79E-02	6.71E-01
Tfh	BCL6	1.80E-01	7.06E-06	1.81E-01	2.12E-05	2.67E-02	8.30E-01	1.14E-01	3.93E-01	1.57E-01	6.32E-02	8.17E-02	3.59E-01
	IL21	2.58E-02	5.22E-01	−2.08E-02	6.28E-01	−1.41E-02	9.10E-01	-1.75E-01	1.90E-01	8.52E-02	3.17E-01	9.64E-03	9.14E-01
Th17	IL17A	4.95E-02	2.19E-01	1.25E-02	7.71E-01	3.11E-02	8.03E-01	-9.34E-03	9.45E-01	7.50E-02	3.78E-01	2.59E-02	7.72E-01
Treg	FOXP3	1.48E-01	2.22E-04	6.58E-02	1.25E-01	7.54E-02	5.43E-01	-5.34E-03	9.68E-01	1.86E-01	2.76E-02	6.87E-02	4.41E-01
	CCR8	2.17E-01	5.07E-08	1.62E-01	1.50E-04	2.56E-01	3.70E-02	1.87E-01	1.61E-01	2.94E-01	4.15E-04	2.19E-01	1.32E-02
	STAT5B	2.84E-01	9.22E-13	2.85E-01	1.23E-11	2.75E-01	2.47E-02	1.98E-01	1.37E-01	1.95E-01	2.10E-02	1.64E-01	6.40E-02
	TGFB1	4.13E-01	8.75E-27	3.73E-01	2.13E-19	3.68E-01	2.31E-03	3.14E-01	1.63E-02	4.10E-01	6.09E-07	3.40E-01	8.42E-05
T-cell exhaustion	PDCD1	−1.46E-02	7.18E-01	−1.15E-01	7.21E-03	−1.09E-01	3.80E-01	−2.05E-01	1.22E-01	1.68E-01	1.17E-03	6.26E-01	4.82E-03
	CTLA4	1.07E-01	7.75E-03	3.70E-02	3.91E-01	4.46E-02	7.19E-01	−2.91E-02	8.28E-01	1.21E-01	1.54E-01	5.16E-03	9.54E-01
	HAVCR2	3.55E-01	1.07E-19	3.01E-01	7.59E-13	4.34E-01	2.78E-04	4.12E-01	1.30E-03	3.41E-01	4.27E-05	2.92E-01	8.22E-04
	GZMB	−2.25E-03	9.56E-01	−1.04E-01	1.55E-02	−1.24E-01	3.16E-01	−2.13E-01	1.09E-01	7.20E-02	3.98E-01	−4.53E-02	6.12E-01

### RAI14 Correlates With CPN1 and PD-L1 in TNBC Cell Lines

**FIGURE 7:**
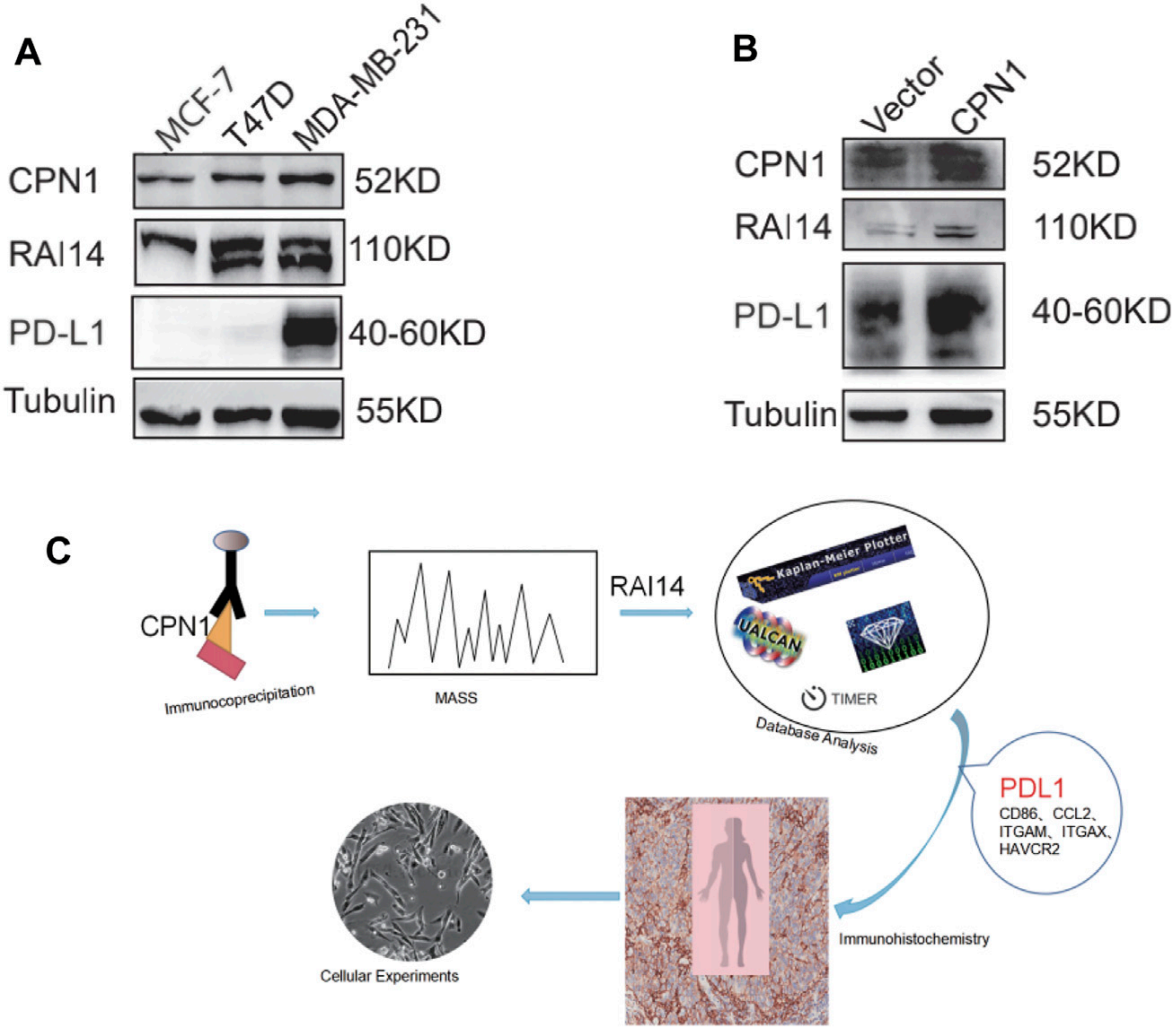
In TNBC cell lines, CPN1 positively correlated with RAI14 and PDL1 expression. **(A)** Expression levels of CPN1, RAI14, and PDL1 in breast cancer cell lines. **(B)** Increased expression of RAI14 and PDL1 in MDA-MB-231 cell lines with overexpression of CPN1. **(C)** Our protocol summarized in a schematic diagram.

We examine the expression of CPN1 and RAI14 using the Western blot in different mammary carcinoma cell lines. As shown in Figure 7A, CPN1 and RAI14 were expressed in triple-negative breast cancer cell lines and were positively correlated. Interestingly, PD-L1 was only expressed in MDA-MB-231 with the TNBC cell line. The expression of RAI14 and PD-L1 was also increased accompanied by CPN1 overexpression (Figure 7B). Therefore, these results suggested that CPN1 shows a positive relationship with the expression of RAI14 and PD-L1. In addition, RAI14 levels in TNBC were related to the level of immune cell-related genes and markers. Our aforementioned findings were further validated by histochemical staining and cellular assays (Figure 7C).

## Discussion

The retinoic acid-inducible protein 14 (RAI14),described as a marker for retinal pigment endothelial cells, acts as a developmental regulator (Kutty et al., 2001). It was shown that RAI14 is expressed in many human tissues, especially in human placental and testicular tissues, and its function is tightly linked to the cellular skeleton (Peng et al., 2000; Yuan et al., 2005). Nowadays, accumulated studies focus on the role of RAI14 in the oncology field. In past decades, several research studies have shown that RAI14 was higher in a wide range of malignancies, containing esophageal cancer (Wang et al., 2020b), gastric cancer (Chen et al., 2018; He et al., 2018; Meng et al., 2020; Xiao et al., 2020), lung adenocarcinoma (Yuan et al., 2017), ovarian cancer (Hawkins et al., 2013), and prostate cancer (Paez et al., 2016), and positively related to the progression of neoplasms.

Previous studies suggested that CPN1 and RAI14 may be reciprocal proteins. CPN1 is localized intracellularly and secreted through HPA (https://www.proteinatlas.org/) and Genecard (www.genecards.org), we therefore discovered that cytosolic CPN1 interacts with intracellular proteins, such as RAI14. Furthermore, cytological experiments were conducted to validate that CPN1 may serve an important part in aggression and metastasis of breast cancer (Cui et al., 2016). Also, the results from serological experiments indicated that the serum CPN1 may be a potential biomarker comparedwith CA15-3 for breast cancer (Cui et al., 2020). The high-RAI14 level of these malignancies is remarkably in contrast to the resistance reaction to the aggression of medications and neoplastic cells. Nevertheless, RAI14 levels and clinical prognosis in TNBC have not been investigated.

In the current investigation, a comprehensive bioinformatics-based analysis uncovered this linkage between RAI14 expression and prognosis of each subset, and also predicted the relationship between RAI14 expression and the level of immune infiltration for immune cells in TNBC. Based on GEPIA2.0 and TIMER database bioinformatics analysis, the mRNA expression of RAI14 was highly expressed in breast cancerous tissues and TNBC relative to normal breast tissues. RAI14 expression and prognosis were correlated with the subclass, histological type, cancer stage, menopause status, age, estrogen receptor (ER), and triple-negative status (Supplementary Table S1 and Supplementary Figure S3). The information from the Bc-GenExMinerv4.7 database indicated that RAI14 mRNA expression was highly expressed in ER-breast cancer and TNBC. In addition, patients with high RAI14 expression and ER+ had higher OS [HR = 0.82, 95% CI (0.70, 0.96), *p* = 0.0135] than those with low RAI14 expression (Supplementary Figure S1B). According to Kaplan–Meier analysis, TNBC patients with high RAI14 expression had poorer survival time (*p* = 0.015). From the ROC, it is known that RAI14 expression had a strong predictive potential on the prognosis of patients with an AUC of 0.736 (Supplementary Figure S1A). Also, the expression of RAI14 was significantly different among different disease stages and tumor grading. These results suggested that RAI14 expression may influence the course of TNBC and may serve as an independent biomarker of poor prognosis in TNBC. In other words, RAI14 expression may be involved in the progression of TNBC and could have a prospecting value as a progressive marker for TNBC. Also, RAI14 is a promising potential therapeutic target for cancer therapy.

Moreover, our achievements demonstrated that the expression of RAI14 related to the expression of infiltrating immune cells markers in TNBC. It showed that the relevance between RAI14 expression and markers of infiltrating immune cells in TNBC. Our outcomes showed that RAI14 expression is positively associated with macrophages and neutrophils (Supplementary Figure S4), and thereby demonstrated that RAI14 expression is related to immune infiltration in the tumor microenvironment of TNBC, where it positively correlated with T-cell exhaustion, macrophage, tumor-associated macrophages (TAMs), dendritic cell, monocyte, and neutrophil infiltration. This relationship between RAI14 and immune infiltration suggested that they were possibly relevant to cancer oncogenesis and development. Infiltrative and immune cells in the tumor microenvironment may influence the survival of tumor patients through direct or indirect participation in both the immune response and angiogenesis (Jiang et al., 2017). It is known that dendritic cells can capture and present antigens released by tumor cells (Wu et al., 2004). Also, the distribution and transcriptome of monocyte subpopulations are significant variations in the presence of endometrial and breast cancers (Cassetta et al., 2019). The aforementioned findings suggest that cancers are linked to infiltrating immune cells in the tumor microenvironment, which can significantly affect treatment efficacy and the overall survival rate.

The correlation between macrophage infiltration and malignancy is a topical topic in current investigations. Macrophages are known to be multipurpose cells which can polarize into inflammation-promoting macrophage M1 and immunosuppressive macrophage M2 under the stimulation of differential chemotactic factors (Murray and Wynn, 2011). Both M1 and M2 are involved with the process of cancer prognosis, but M1 primarily contributes to the protective role by triggering the production of toxic mediator and reactive oxygen intermediates, whereas M2 acts as a risk factor for cancer progression (Hellstrand, 2003). Tumor-associated macrophages (TAMs) are highly compatible with M2-polarized macrophages, and it has been reported that TAMs can induce angiogenesis to assist tumor invasion, shape breast cancer cells that have escaped the host immune system, and recruit immunosuppressive leukocytes to the tumor microenvironment. Thus, the accumulation of TAMs in breast cancer leads to poor patient prognosis (Choi et al., 2018). It is reasonable to propose that RAI14 may interact with macrophages and lead to TNBC progression. However, this hypothesis remains to be validated by further studies.

In addition, RAI14 expression is significantly related to T-cell exhaustion markers such as PD1. It is well known that PD1 and PD-L1 are important immune checkpoint components, since they modulate the function of tumor cells and tumor-infiltrating lymphocytes (TILs) (Baba et al., 2020). Schalper et al. have reported that 58% of breast cancer specimens had PD-L1 mRNA expression (Schalper et al., 2014). In addition, Ghebeh et al. reported a correlation between PD-L1 expression and advanced tumor grade, patient age, P53 mutation status, menopause status, and ER expression, which is consistent with our results; Nevertheless, no major association was found with lymph node status and PR expression (Ghebeh et al., 2006). It has reported the most powerful association between PD-L1 and decreased survival in TNBC (Soliman et al., 2014), and more importantly, and the PD1 and PD-L1 has been shown to be a proactive therapy target for invasive breast cancer (Bertucci et al., 2016). Similarly, S. Muenst et al. suggested that PD-L1 expression in breast cancer specimens is associated with larger tumor size, more advanced tumor process, and a positive lymph node status, which may suggest that involvement of PD-1/PD-L1 pathway may contribute to the immune escape, thereby influencing the rate of proliferation and spreading more rapidly (Muenst et al., 2014). Moreover, it has shown that PD-1 may have a significant function in the response to tumor oppositions (Woo et al., 2012).

Herein, we also verified that CPN1 overexpression in TNBC cell lines is accompanied by an upregulated expression level of RAI14 and PD-L1 through histochemical and cytological experiments, suggesting a correlation among them (Figures 6, 7). Our results indicated that RAI14 may be a key downstream molecule of CPN1, which controls immune cell recruitment to TNBC and plays essential functions of regulating infiltrating immune cells, and thus RAI14 acts as a potential prognostic biomarker in TNBC. These correlations may suggest potential mechanisms by which RAI14 regulates T-cell function. Consequently, such discoveries indicate that RAI14 expression is associated with the level of immune infiltration in breast cancer, particularly TNBC, and provides a direction for breast cancer treatment in clinical settings.

## Conclusion

Overall, high expression of RAI14 denotes poor prognosis in TNBC accompanied by increasing levels of immune infiltration of macrophages, dendritic cells, monocytes, and neutrophils and PD1, implying that the role of RAI14 in regulating immune cell infiltration in the TME may provide novel insights into TNBC progression and metastasis.

## Data Availability

The datasets presented in this study can be found in online repositories. The names of the repository/repositories and accession number(s) can be found in the article/Supplementary Material.

## Abbreviations

RAI14, retinoic acid-induced protein 14; CPN1, carboxypeptidase N1; CO-IP, co-immunoprecipitation; TIMER, Tumor Immune Estimation Resource; HPA, Human Protein Atlas; GEPIA, Gene Expression Profiling Interaction Analysis BC-GenExMiner Breast Cancer Gene-Expression Miner; ER, estrogen receptor; TNBC, triple-negative breast cancer; CTLA4, cytotoxic T lymphocyte-associated antigen 4; PD-1, programmed cell death 1; PD-L1, programmed death ligand-1; TAMs, tumor-associated aacrophages; TINs, tumor-infiltrating neutrophils; TCGA, The Cancer Genome Atlas; OS, overall survival; RFS, relapse-free survival; DFS, disease-free survival; SDS-PAGE, sulfate–polyacrylamide gel electrophoresis; ILC, invasive lobular carcinoma; IDC, invasive ductal carcinoma; NORPEG, novel retinal pigment endothelial cell gene; ARPE-19, retinal pigment epithelial cells; TILs, tumor-infiltrating lymphocytes.

## References

[B1] Alsaleem M. A. Ball G. Toss M. S. Raafat S. Aleskandarany M. Joseph C. (2020). A Novel Prognostic Two-Gene Signature for Triple Negative Breast Cancer. Mod. Pathol. 33 (11), 2208–2220. 10.1038/s41379-020-0563-7 32404959

[B2] Baba Y. Nomoto D. Okadome K. Ishimoto T. Iwatsuki M. Miyamoto Y. (2020). Tumor Immune Microenvironment and Immune Checkpoint Inhibitors in Esophageal Squamous Cell Carcinoma. Cancer Sci. 111 (9), 3132–3141. 10.1111/cas.14541 32579769 PMC7469863

[B3] Bertucci F. Finetti P. Birnbaum D. Mamessier E. (2016). The PD1/PDL1 axis, a Promising Therapeutic Target in Aggressive Breast Cancers. Oncoimmunology 5 (3), e1085148. 10.1080/2162402X.2015.1085148 27141340 PMC4839307

[B4] Billon E. Finetti P. Bertucci A. Niccoli P. Birnbaum D. Mamessier E. (2019). PDL1 Expression Is Associated with Longer Postoperative, Survival in Adrenocortical Carcinoma. Oncoimmunology 8 (11), e1655362. 10.1080/2162402X.2019.1655362 31646101 PMC6791455

[B5] Björk Gunnarsdottir F. Auoja N. Bendahl P. O. Rydén L. Fernö M. Leandersson K. (2020). Co-localization of CD169+ Macrophages and Cancer Cells in Lymph Node Metastases of Breast Cancer Patients Is Linked to Improved Prognosis and PDL1 Expression. Oncoimmunology 9 (1), 1848067. 10.1080/2162402X.2020.1848067 33299660 PMC7714471

[B6] Bour-Jordan H. Esensten J. H. Martinez-Llordella M. Penaranda C. Stumpf M. Bluestone J. A. (2011). Intrinsic and Extrinsic Control of Peripheral T-Cell Tolerance by Costimulatory Molecules of the CD28/B7 Family. Immunol. Rev. 241 (1), 180–205. 10.1111/j.1600-065X.2011.01011.x 21488898 PMC3077803

[B7] Cassetta L. Fragkogianni S. Sims A. H. Swierczak A. Forrester L. M. Zhang H. (2019). Human Tumor-Associated Macrophage and Monocyte Transcriptional Landscapes Reveal Cancer-specific Reprogramming, Biomarkers, and Therapeutic Targets. Cancer cell 35 (4), 588–e10. 10.1016/j.ccell.2019.02.009 30930117 PMC6472943

[B8] Chen C. Maimaiti A. Zhang X. Qu H. Sun Q. He Q. (2018). Knockdown of RAI14 Suppresses the Progression of Gastric Cancer. Onco Targets Ther. 11, 6693–6703. 10.2147/OTT.S175502 30349303 PMC6186306

[B9] Choi J. Gyamfi J. Jang H. Koo J. S. (2018). The Role of Tumor-Associated Macrophage in Breast Cancer Biology. Histol. Histopathol 33 (2), 133–145. 10.14670/HH-11-916 28681373

[B10] Colwill K. au fnm. Gräslund S. (2011). A Roadmap to Generate Renewable Protein Binders to the Human Proteome. Nat. Methods 8 (7), 551–558. 10.1038/nmeth.1607 21572409

[B11] Cui R. Wang C. Li T. Hua J. Zhao T. Ren L. (2021). Carboxypeptidase N1 Is Anticipated to Be a Synergy Metrics for Chemotherapy Effectiveness and Prognostic Significance in Invasive Breast Cancer. Cancer Cel Int 21 (1), 571. 10.1186/s12935-021-02256-5 PMC8555242 34711246

[B12] Cui R. Wang C. Zhao Q. Wang Y. Li Y. (2020). Serum Carboxypeptidase N1 Serves as a Potential Biomarker Complementing CA15-3 for Breast Cancer. Anticancer Agents Med. Chem. 20 (17), 2053–2065. 10.2174/1871520620666200703191135 32619179

[B13] Cui R. Zhang P. Li Y. (2016). Role of Carboxypeptidase N Invasion and Migration in Breast Cancer. Anticancer Agents Med. Chem. 16 (9), 1198–1202. 10.2174/1871520616666160201104939 26860443

[B14] de Andrea C. E. Schalper K. A. Sanmamed M. F. Melero I. (2018). Immunodivergence in Metastatic Colorectal Cancer. Cancer cell 34 (6), 876–878. 10.1016/j.ccell.2018.11.012 30537510 PMC7385539

[B15] Dong H. Strome S. E. Salomao D. R. Tamura H. Hirano F. Flies D. B. (2002). Tumor-associated B7-H1 Promotes T-Cell Apoptosis: a Potential Mechanism of Immune Evasion. Nat. Med. 8 (8), 793–800. 10.1038/nm730 12091876

[B16] Duan X. Chan C. Guo N. Han W. Weichselbaum R. R. Lin W. (2016). Photodynamic Therapy Mediated by Nontoxic Core-Shell Nanoparticles Synergizes with Immune Checkpoint Blockade to Elicit Antitumor Immunity and Antimetastatic Effect on Breast Cancer. J. Am. Chem. Soc. 138 (51), 16686–16695. 10.1021/jacs.6b09538 27976881 PMC5667903

[B17] Ghebeh H. Mohammed S. Al-Omair A. Qattan A. Lehe C. Al-Qudaihi G. (2006). The B7-H1 (PD-L1) T Lymphocyte-Inhibitory Molecule Is Expressed in Breast Cancer Patients with Infiltrating Ductal Carcinoma: Correlation with Important High-Risk Prognostic Factors. Neoplasia 8 (3), 190–198. 10.1593/neo.05733 16611412 PMC1578520

[B18] Ham S. W. Jeon H. Y. Jin X. Kim E. J. Kim J. K. Shin Y. J. (2019). TP53 Gain-Of-Function Mutation Promotes Inflammation in Glioblastoma. Cell Death Differ 26 (3), 409–425. 10.1038/s41418-018-0126-3 29786075 PMC6370770

[B19] Hawkins S. M. Loomans H. A. Wan Y. W. Ghosh-Choudhury T. Coffey D. Xiao W. (2013). Expression and Functional Pathway Analysis of Nuclear Receptor NR2F2 in Ovarian Cancer. J. Clin. Endocrinol. Metab. 98 (7), E1152–E1162. 10.1210/jc.2013-1081 23690307 PMC3701283

[B20] He X. Y. Zhao J. Chen Z. Q. Jin R. Liu C. Y. (2018). High Expression of Retinoic Acid Induced 14 (RAI14) in Gastric Cancer and its Prognostic Value. Med. Sci. Monit. 24, 2244–2251. 10.12659/msm.910133 29654694 PMC5912095

[B21] Hellstrand K. (2003). Melanoma Immunotherapy: a Battle against Radicals? Trends Immunol. 24 (5), 232–234. 10.1016/s1471-4906(03)00070-x 12738414

[B22] Iwai Y. Ishida M. Tanaka Y. Okazaki T. Honjo T. Minato N. (2002). Involvement of PD-L1 on Tumor Cells in the Escape from Host Immune System and Tumor Immunotherapy by PD-L1 Blockade. Proc. Natl. Acad. Sci. U S A. 99 (19), 12293–12297. 10.1073/pnas.192461099 12218188 PMC129438

[B23] Jiang W. Liu K. Guo Q. Cheng J. Shen L. Cao Y. (2017). Tumor-infiltrating Immune Cells and Prognosis in Gastric Cancer: a Systematic Review and Meta-Analysis. Oncotarget 8 (37), 62312–62329. 10.18632/oncotarget.17602 28977947 PMC5617507

[B24] Jin X. Xie H. Liu X. Shen Q. Wang Z. Hao H. (2020). RELL1, a Novel Oncogene, Accelerates Tumor Progression and Regulates Immune Infiltrates in Glioma. Int. Immunopharmacol 87, 106707. 10.1016/j.intimp.2020.106707 32683297

[B25] Keir M. E. Butte M. J. Freeman G. J. Sharpe A. H. (2008). PD-1 and its Ligands in Tolerance and Immunity. Annu. Rev. Immunol. 26, 677–704. 10.1146/annurev.immunol.26.021607.090331 18173375 PMC10637733

[B26] Kutty R. K. Kutty G. Samuel W. Duncan T. Bridges C. C. El-Sherbeeny A. (2001). Molecular Characterization and Developmental Expression of NORPEG, a Novel Gene Induced by Retinoic Acid. J. Biol. Chem. 276 (4), 2831–2840. 10.1074/jbc.M007421200 11042181

[B27] Li T. Fu J. Zeng Z. Cohen D. Li J. Chen Q. (2020). TIMER2.0 for Analysis of Tumor-Infiltrating Immune Cells. Nucleic Acids Res. 48 (W1), W509–W514. 10.1093/nar/gkaa407 32442275 PMC7319575

[B28] Meng C. Xia S. He Y. Tang X. Zhang G. Zhou T. (2020). Discovery of Prognostic Signature Genes for Overall Survival Prediction in Gastric Cancer. Comput. Math. Methods Med. 2020, 5479279. 10.1155/2020/5479279 32908579 PMC7468614

[B29] Muenst S. Schaerli A. R. Gao F. Däster S. Trella E. Droeser R. A. (2014). Expression of Programmed Death Ligand 1 (PD-L1) Is Associated with Poor Prognosis in Human Breast Cancer. Breast Cancer Res. Treat. 146 (1), 15–24. 10.1007/s10549-014-2988-5 24842267 PMC4180714

[B30] Murray P. J. Wynn T. A. (2011). Protective and Pathogenic Functions of Macrophage Subsets. Nat. Rev. Immunol. 11 (11), 723–737. 10.1038/nri3073 21997792 PMC3422549

[B31] Niknam S. Barsoumian H. B. Schoenhals J. E. Jackson H. L. Yanamandra N. Caetano M. S. (2018). Radiation Followed by OX40 Stimulation Drives Local and Abscopal Antitumor Effects in an Anti-PD1-resistant Lung Tumor Model. Clin. Cancer Res. 24 (22), 5735–5743. 10.1158/1078-0432.CCR-17-3279 29784675 PMC6239963

[B32] Paez A. V. Pallavicini C. Schuster F. Valacco M. P. Giudice J. Ortiz E. G. (2016). Heme Oxygenase-1 in the Forefront of a Multi-Molecular Network that Governs Cell-Cell Contacts and Filopodia-Induced Zippering in Prostate Cancer. Cell Death Dis 7 (12), e2570. 10.1038/cddis.2016.420 28032857 PMC5261017

[B33] Peng Q. Wen T. Liu D. Wang S. Jiang X. Zhao S. (2021). DSN1 Is a Prognostic Biomarker and Correlated with Clinical Characterize in Breast Cancer. Int. Immunopharmacol 101, 107605. 10.1016/j.intimp.2021.107605 34238686

[B34] Peng Y. F. Mandai K. Sakisaka T. Okabe N. Yamamoto Y. Yokoyama S. (2000). Ankycorbin: a Novel Actin Cytoskeleton-Associated Protein. Genes Cells 5 (12), 1001–1008. 10.1046/j.1365-2443.2000.00381.x 11168586

[B35] Schalper K. A. Velcheti V. Carvajal D. Wimberly H. Brown J. Pusztai L. (2014). *In Situ* tumor PD-L1 mRNA Expression Is Associated with Increased TILs and Better Outcome in Breast Carcinomas. Clin. Cancer Res. 20 (10), 2773–2782. 10.1158/1078-0432.CCR-13-2702 24647569

[B36] Soliman H. Khalil F. Antonia S. (2014). PD-L1 Expression Is Increased in a Subset of Basal Type Breast Cancer Cells. PloS one 9 (2), e88557. 10.1371/journal.pone.0088557 24551119 PMC3925108

[B37] Stamm H. Oliveira-Ferrer L. Grossjohann E. M. Muschhammer J. Thaden V. Brauneck F. (2019). Targeting the TIGIT-PVR Immune Checkpoint axis as Novel Therapeutic Option in Breast Cancer. Oncoimmunology 8 (12), e1674605. 10.1080/2162402X.2019.1674605 31741778 PMC6844319

[B38] Tang Z. Li C. Kang B. Gao G. Li C. Zhang Z. (2017). GEPIA: a Web Server for Cancer and normal Gene Expression Profiling and Interactive Analyses. Nucleic Acids Res. 45 (W1), W98–W102. 10.1093/nar/gkx247 28407145 PMC5570223

[B39] Wang J. Cai Y. Luo J. Sun Z. Yu J. Yan F. (2020). RAI14 Silencing Suppresses Progression of Esophageal Cancer via the STAT3 Pathway. Aging (Albany NY) 12 (18), 18084–18098. 10.18632/aging.103613 32957082 PMC7585088

[B40] Wang Z. Jiang Q. Dong C. (2020). Metabolic Reprogramming in Triple-Negative Breast Cancer. Cancer Biol. Med. 17 (1), 44–59. 10.20892/j.issn.2095-3941.2019.0210 32296576 PMC7142847

[B41] Waniczek D. Lorenc Z. Śnietura M. Wesecki M. Kopec A. Muc-Wierzgoń M. (2017). Tumor-Associated Macrophages and Regulatory T Cells Infiltration and the Clinical Outcome in Colorectal Cancer. Arch. Immunol. Ther. Exp. (Warsz) 65 (5), 445–454. 10.1007/s00005-017-0463-9 28343267 PMC5602054

[B42] Woo S. R. Turnis M. E. Goldberg M. V. Bankoti J. Selby M. Nirschl C. J. (2012). Immune Inhibitory Molecules LAG-3 and PD-1 Synergistically Regulate T-Cell Function to Promote Tumoral Immune Escape. Cancer Res. 72 (4), 917–927. 10.1158/0008-5472.CAN-11-1620 22186141 PMC3288154

[B43] Wu Y. Wang L. Zhang Y. (2004). Dendritic Cells as Vectors for Immunotherapy of Tumor and its Application for Gastric Cancer Therapy. Cell Mol Immunol 1 (5), 351–356. 16285894

[B44] Xiao Y. Zhang H. Du G. Meng X. Wu T. Zhou Q. (2020). RAI14 Is a Prognostic Biomarker and Correlated with Immune Cell Infiltrates in Gastric Cancer. Technol. Cancer Res. Treat. 19, 1533033820970684. 10.1177/1533033820970684 33176601 PMC7672724

[B45] Yang Z. Li J. Feng G. Gao S. Wang Y. Zhang S. (2017). MicroRNA-145 Modulates N6-Methyladenosine Levels by Targeting the 3'-Untranslated mRNA Region of the N6-Methyladenosine Binding YTH Domain Family 2 Protein. J. Biol. Chem. 292 (9), 3614–3623. 10.1074/jbc.M116.749689 28104805 PMC5339747

[B46] Yuan C. Hu H. Kuang M. Chen Z. Tao X. Fang S. (2017). Super Enhancer Associated RAI14 Is a New Potential Biomarker in Lung Adenocarcinoma. Oncotarget 8 (62), 105251–105261. 10.18632/oncotarget.22165 29285248 PMC5739635

[B47] Yuan W. Zheng Y. Huo R. Lu L. Huang X. Y. Yin L. L. (2005). Expression of a Novel Alternative Transcript of the Novel Retinal Pigment Epithelial Cell Gene NORPEG in Human Testes. Asian J. Androl. 7 (3), 277–288. 10.1111/j.1745-7262.2005.00040.x 16110356

[B48] Zhang H. Liu H. Shen Z. Lin C. Wang X. Qin J. (2018). Tumor-infiltrating Neutrophils Is Prognostic and Predictive for Postoperative Adjuvant Chemotherapy Benefit in Patients with Gastric Cancer. Ann. Surg. 267 (2), 311–318. 10.1097/SLA.0000000000002058 27763900

[B49] Zhang J. Zhang Q. Zhang J. Wang Q. (2020). Expression of ACAP1 Is Associated with Tumor Immune Infiltration and Clinical Outcome of Ovarian Cancer. DNA Cel Biol 39 (9), 1545–1557. 10.1089/dna.2020.5596 32456571

[B50] Zhang M. Chen H. Wang M. Bai F. Wu K. (2020). Bioinformatics Analysis of Prognostic Significance of COL10A1 in Breast Cancer. Biosci. Rep. 40 (2). 10.1042/BSR20193286 PMC7029149 32043519

[B51] Zhang Y. Wang S. Yang B. Lu S. Du Y. Liu H. (2019). Adjuvant Treatment for Triple-Negative Breast Cancer: a Retrospective Study of Immunotherapy with Autologous Cytokine-Induced Killer Cells in 294 Patients. Cancer Biol. Med. 16 (2), 350–360. 10.20892/j.issn.2095-3941.2018.0378 31516755 PMC6713632

