# Supplementary material for: High Expression of RAI14 in Triple-Negative Breast Cancer Participates in Immune Recruitment and Implies Poor Prognosis Through Bioinformatics Analyses
Source: Front Pharmacol. 2022 Apr 1;13:809454. doi: 10.3389/fphar.2022.809454 (PMC9010950; doi:10.3389/fphar.2022.809454)
Supplement: Supplementary file 1 [file DataSheet2.PDF]

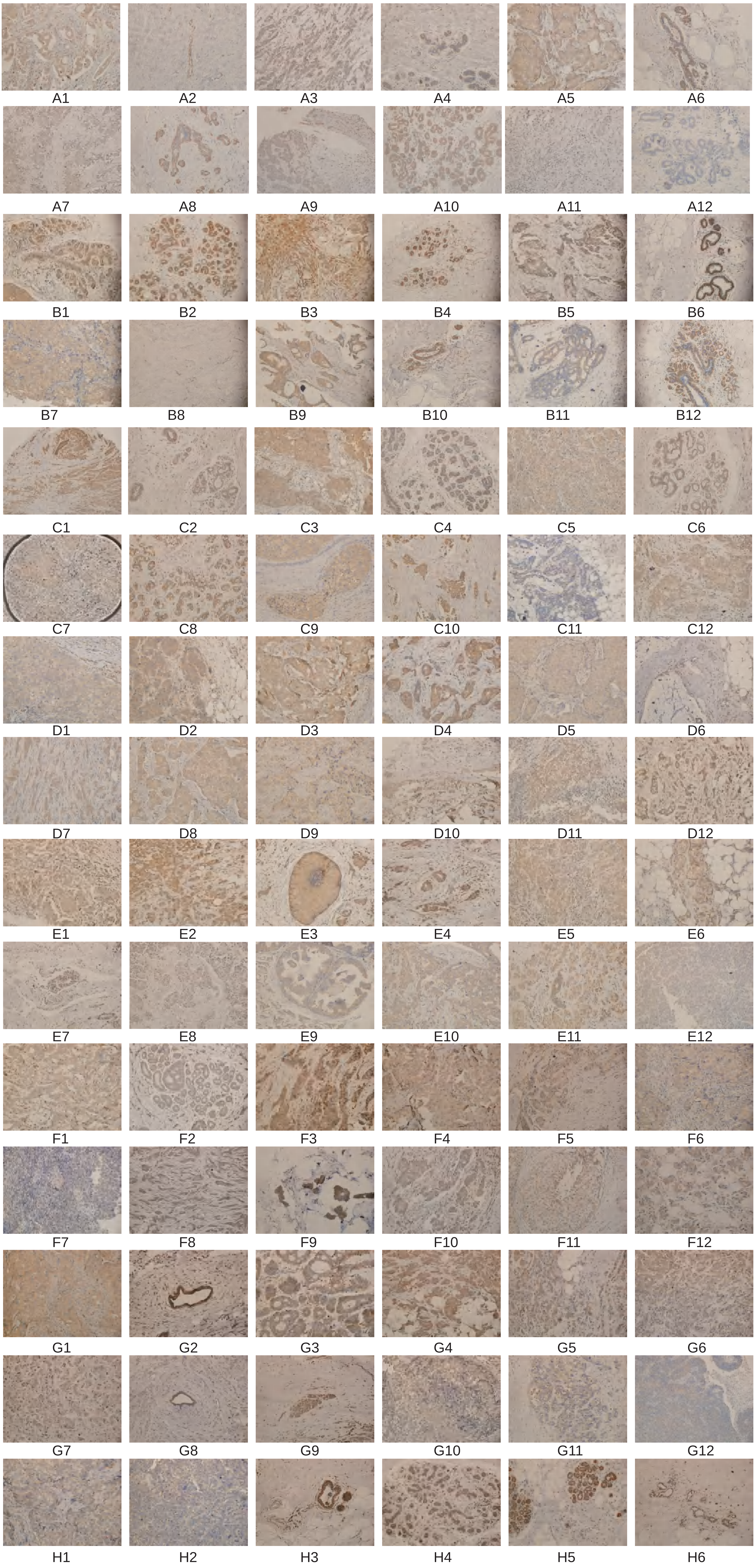
A1 A2 A3 A4 A5 A6
A7 A8 A9 A10 A11 A12
B1 B2 B3 B4 B5 B6
B7 B8 B9 B10 B11 B12
C1 C2 C3 C4 C5 C6
C7 C8 C9 C10 C11 C12
D1 D2 D3 D4 D5 D6
D7 D8 D9 D10 D11 D12
E1 E2 E3 E4 E5 E6
E7 E8 E9 E10 E11 E12
F1 F2 F3 F4 F5 F6
F7 F8 F9 F10 F11 F12
G1 G2 G3 G4 G5 G6
G7 G8 G9 G10 G11 G12
H1 H2 H3 H4 H5 H6
