# Supplementary material for: High Expression of RAI14 in Triple-Negative Breast Cancer Participates in Immune Recruitment and Implies Poor Prognosis Through Bioinformatics Analyses
Source: Front Pharmacol. 2022 Apr 1;13:809454. doi: 10.3389/fphar.2022.809454 (PMC9010950; doi:10.3389/fphar.2022.809454)
Supplement: Supplementary file 2 [file DataSheet4.DOC]

**RAI14,As an Reciprocal Protein of Carboxypeptidase N1,**

**Is a Prognostic Biomarker Associated With Immune Inﬁltration**

**in Triple Negative Breast Cancer**

**A table of contents**

**Contents**

**Supplementary Materials & Methods**

**Supplementary Table**

**Supplementary Figure**

**Supplementary Materials & Methods**

***Clinical Baseline Information Form，ROC curve and Forest charts*** ***of RAI14***

Based on TCGA database, software R (version 3.6) for statistical analysis and visualization, human species.

***Invasive tumor immune cell analysis***

Timer2.0(https://cistrome.shinyapps.io/timer/), A Web Server for comprehensive analysis of tumor-infiltrating immune cells .It is an enhanced version that integrates a variety of state-of-the-art algorithms for immune infiltration estimation. These algorithms are applied to the expression profiles of the Cancer Genome Atlas(TCGA), enabling users to explore various associations between immune infiltration and genetic features in TCGA cohorts.1In Correlation module , we have detected the correlation of RAI14 expression in different breast cancer molecular statues (Luminal,Her2,Basal) with immune cell markers to determine the presence of any corresponding immune cell infiltration. That also provides options for partial correlation conditional on tumor purity or age

***Breast Cancer Gene-Expression Miner v4.7***

Breast Cancer Gene-Expression Miner v4.7(http://bcgenex.centregauducheau.fr/)，is a statistical mining tool of published annotated breast cancer transcriptomic data (DNA microarrays [n = 11 359] and RNA-seq [n = 4 712]). It offers the possibility to explore gene-expression of genes of interest in breast cancer.The statistical analyses are grouped in three modules: "correlation", "expression" and "prognostic" (see flowchart). Results are presented in tables, maps and various kinds of plots (pairwise correlation, box and whisker, bee swarm, violin, raincloud, Kaplan-Meier curves). First we selected the sub-module EXPRESSION of ANALYSIS, then we entered RAI14 and selected the metrics we wanted to analyze.

**Table S1 Clinical Baseline Information Form**

| Characteristic | Low expression of RAI14 | High expression of RAI14 | p |
| --- | --- | --- | --- |
| n | 532 | 533 |  |
| T stage, n (%) |  |  | 0.009 |
| T1 | 116 (10.9%) | 159 (15%) |  |
| T2 | 315 (29.7%) | 300 (28.2%) |  |
| T3 | 81 (7.6%) | 56 (5.3%) |  |
| T4 | 18 (1.7%) | 17 (1.6%) |  |
| N stage, n (%) |  |  | 0.003 |
| N0 | 243 (23.2%) | 264 (25.2%) |  |
| N1 | 184 (17.6%) | 165 (15.8%) |  |
| N2 | 44 (4.2%) | 72 (6.9%) |  |
| N3 | 47 (4.5%) | 27 (2.6%) |  |
| M stage, n (%) |  |  | 1.000 |
| M0 | 422 (46.4%) | 467 (51.4%) |  |
| M1 | 10 (1.1%) | 10 (1.1%) |  |
| Pathologic stage, n (%) |  |  | 0.176 |
| Stage I | 79 (7.6%) | 101 (9.7%) |  |
| Stage II | 305 (29.3%) | 301 (28.9%) |  |
| Stage III | 130 (12.5%) | 108 (10.4%) |  |
| Stage IV | 10 (1%) | 8 (0.8%) |  |
| Age, n (%) |  |  | < 0.001 |
| <=60 | 260 (24.4%) | 328 (30.8%) |  |
| >60 | 272 (25.5%) | 205 (19.2%) |  |
| Histological type, n (%) |  |  | < 0.001 |
| Infiltrating Ductal Carcinoma | 345 (36%) | 412 (43%) |  |
| Infiltrating Lobular Carcinoma | 127 (13.2%) | 75 (7.8%) |  |
| PR status, n (%) |  |  | 0.799 |
| Negative | 163 (16%) | 175 (17.2%) |  |
| Indeterminate | 2 (0.2%) | 2 (0.2%) |  |
| Positive | 342 (33.7%) | 332 (32.7%) |  |
| ER status, n (%) |  |  | < 0.001 |
| Negative | 96 (9.4%) | 141 (13.9%) |  |
| Indeterminate | 2 (0.2%) | 0 (0%) |  |
| Positive | 410 (40.3%) | 368 (36.2%) |  |
| HER2 status, n (%) |  |  | 0.178 |
| Negative | 245 (34.2%) | 303 (42.3%) |  |
| Indeterminate | 7 (1%) | 5 (0.7%) |  |
| Positive | 82 (11.4%) | 75 (10.5%) |  |
| PAM50, n (%) |  |  | < 0.001 |
| Normal | 17 (1.6%) | 23 (2.2%) |  |
| LumA | 291 (27.3%) | 260 (24.4%) |  |
| LumB | 109 (10.2%) | 93 (8.7%) |  |
| Her2 | 54 (5.1%) | 28 (2.6%) |  |
| Basal | 61 (5.7%) | 129 (12.1%) |  |
| Menopause status, n (%) |  |  | 0.016 |
| Pre | 94 (9.8%) | 130 (13.6%) |  |
| Peri | 16 (1.7%) | 23 (2.4%) |  |
| Post | 362 (37.9%) | 331 (34.6%) |  |
| radiation_therapy, n (%) |  |  | 0.242 |
| No | 226 (23.3%) | 206 (21.2%) |  |
| Yes | 261 (26.9%) | 279 (28.7%) |  |
| Anatomic neoplasm subdivisions, n (%) |  |  | 0.022 |
| Left | 257 (24.1%) | 296 (27.8%) |  |
| Right | 275 (25.8%) | 237 (22.3%) |  |
| Age, meidan (IQR) | 61 (50, 69) | 56 (48, 65) | < 0.001 |


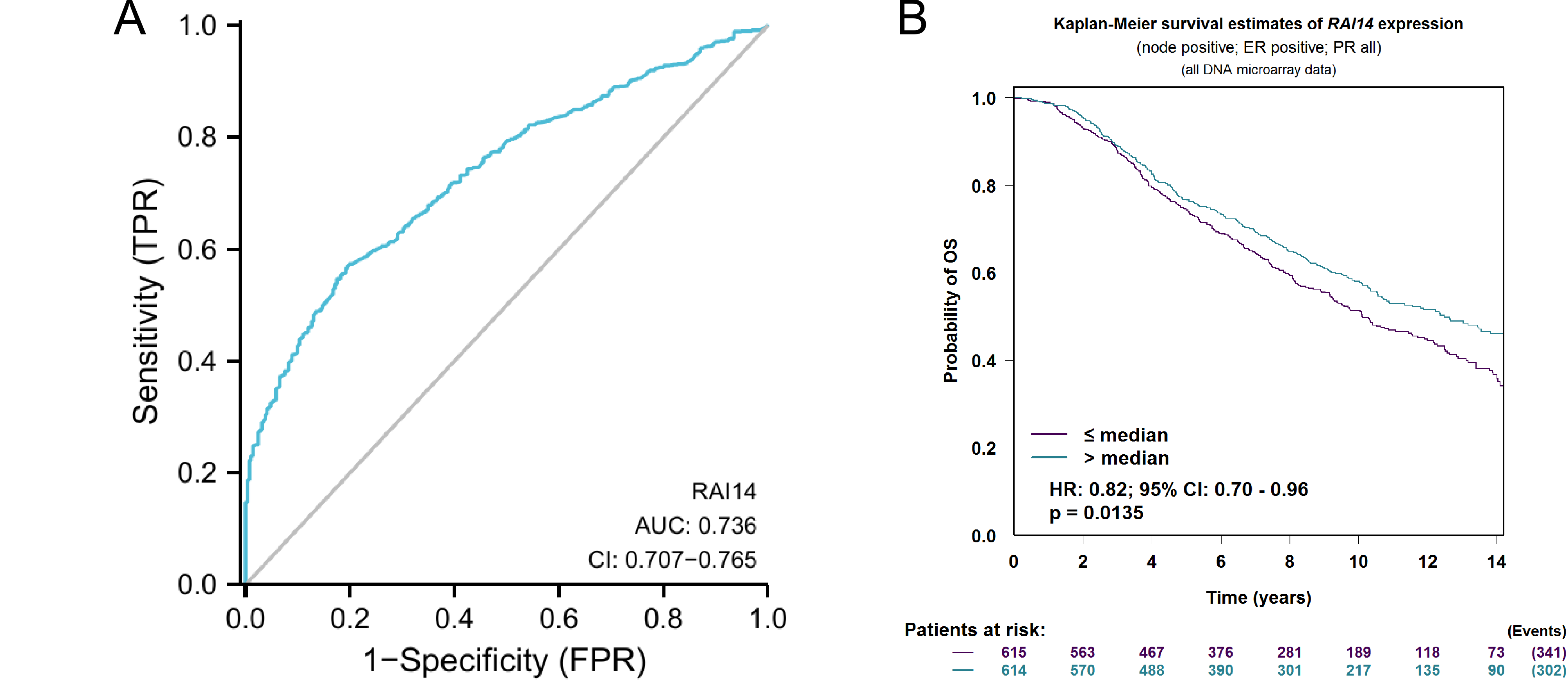


**Figure S1 Diagnostic and prognostic relationship of RAI14 in breast cancer**

**(A) ROC curve of RAI14 (B)** **Association of RAI14 expression with OS(n) in ER-positive breast cancer patients in the GenExMinerv4.6 database**


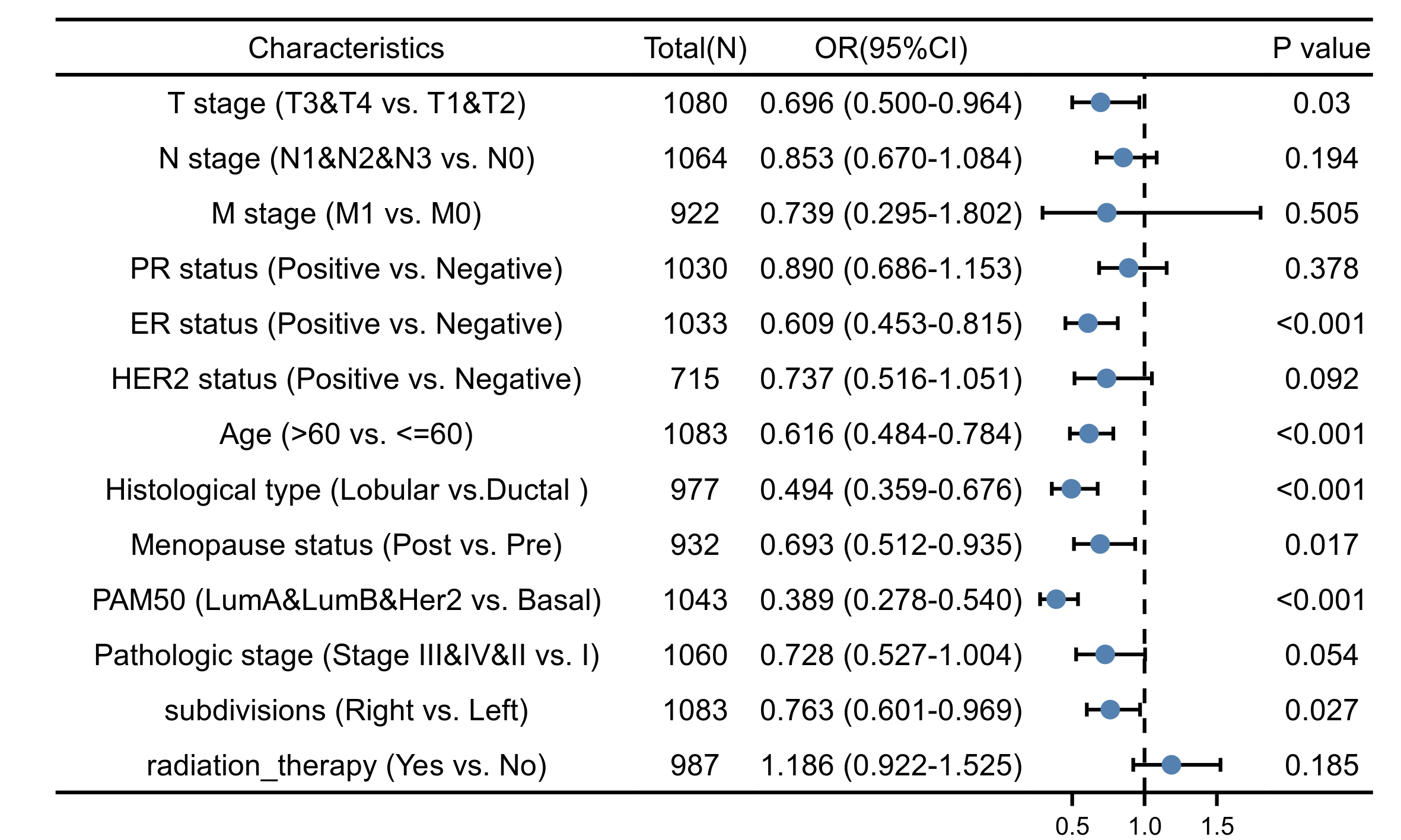


**Figure S2 Forest charts of RAI14**

**
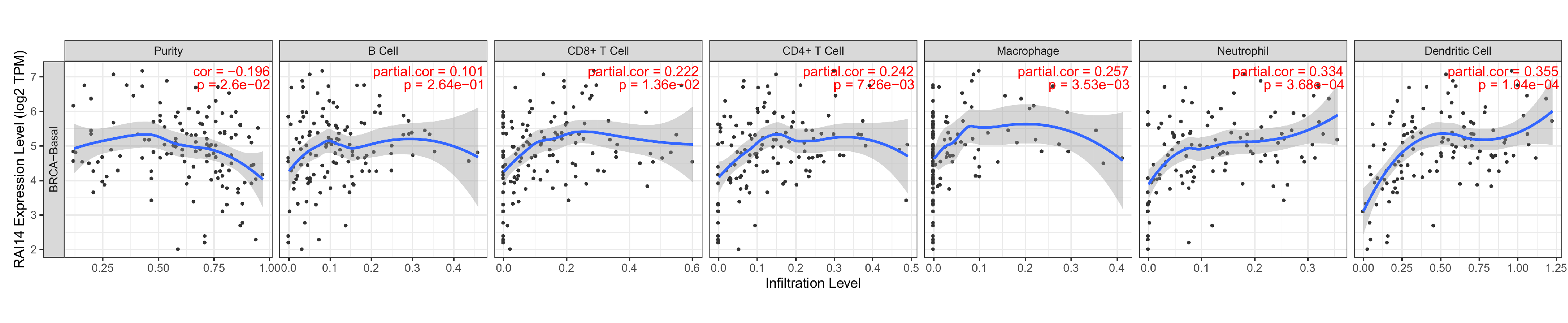
**

**Figure S3 RAI14 Infiltration Level of BRCA-Basal**
