# Supplementary figures and images for: High Expression of RAI14 in Triple-Negative Breast Cancer Participates in Immune Recruitment and Implies Poor Prognosis Through Bioinformatics Analyses
Source: Front Pharmacol. 2022 Apr 1;13:809454. doi: 10.3389/fphar.2022.809454 (PMC9010950; doi:10.3389/fphar.2022.809454)

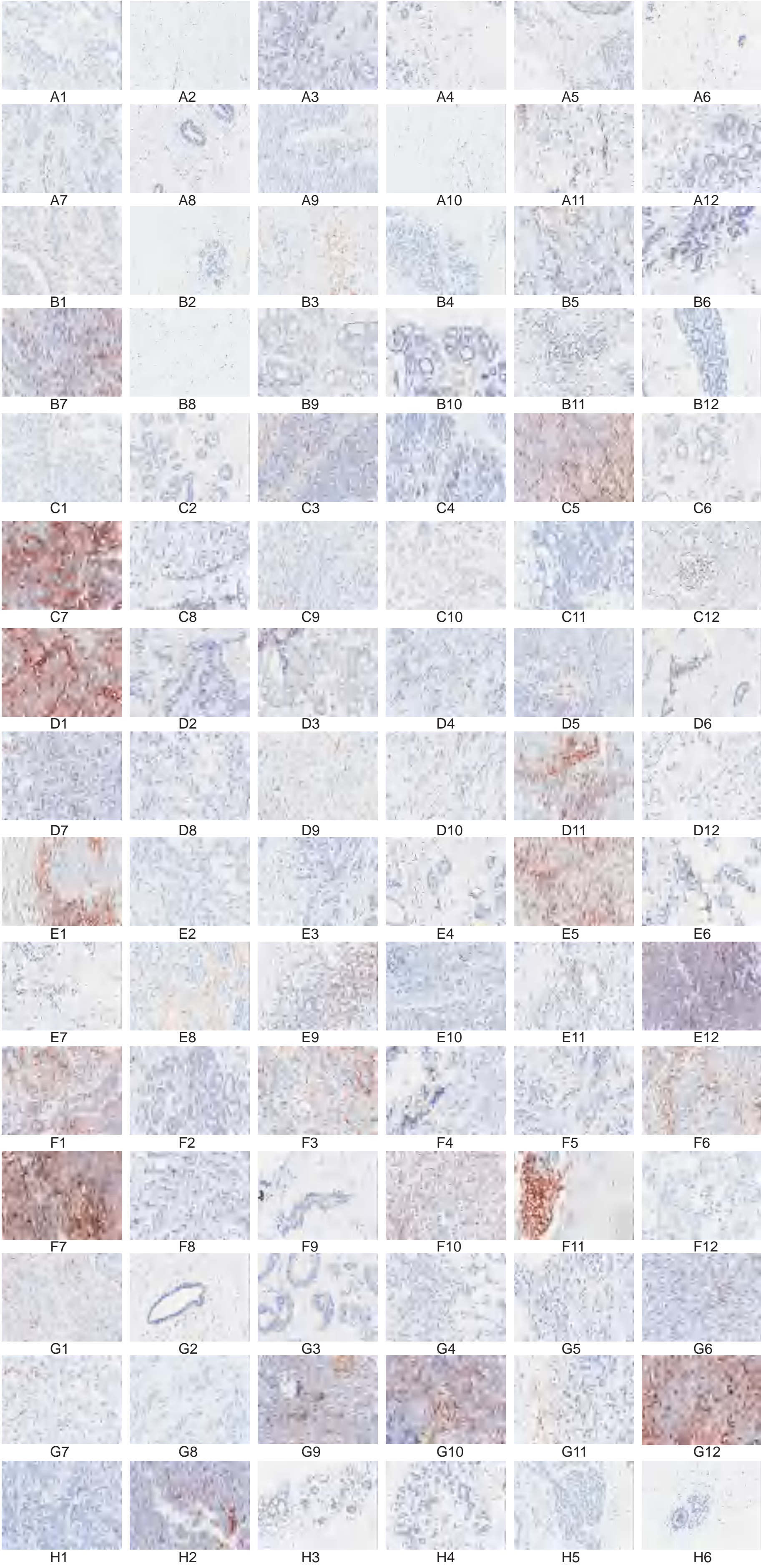

Supplement: Supplementary file 3 [file DataSheet3.PDF]

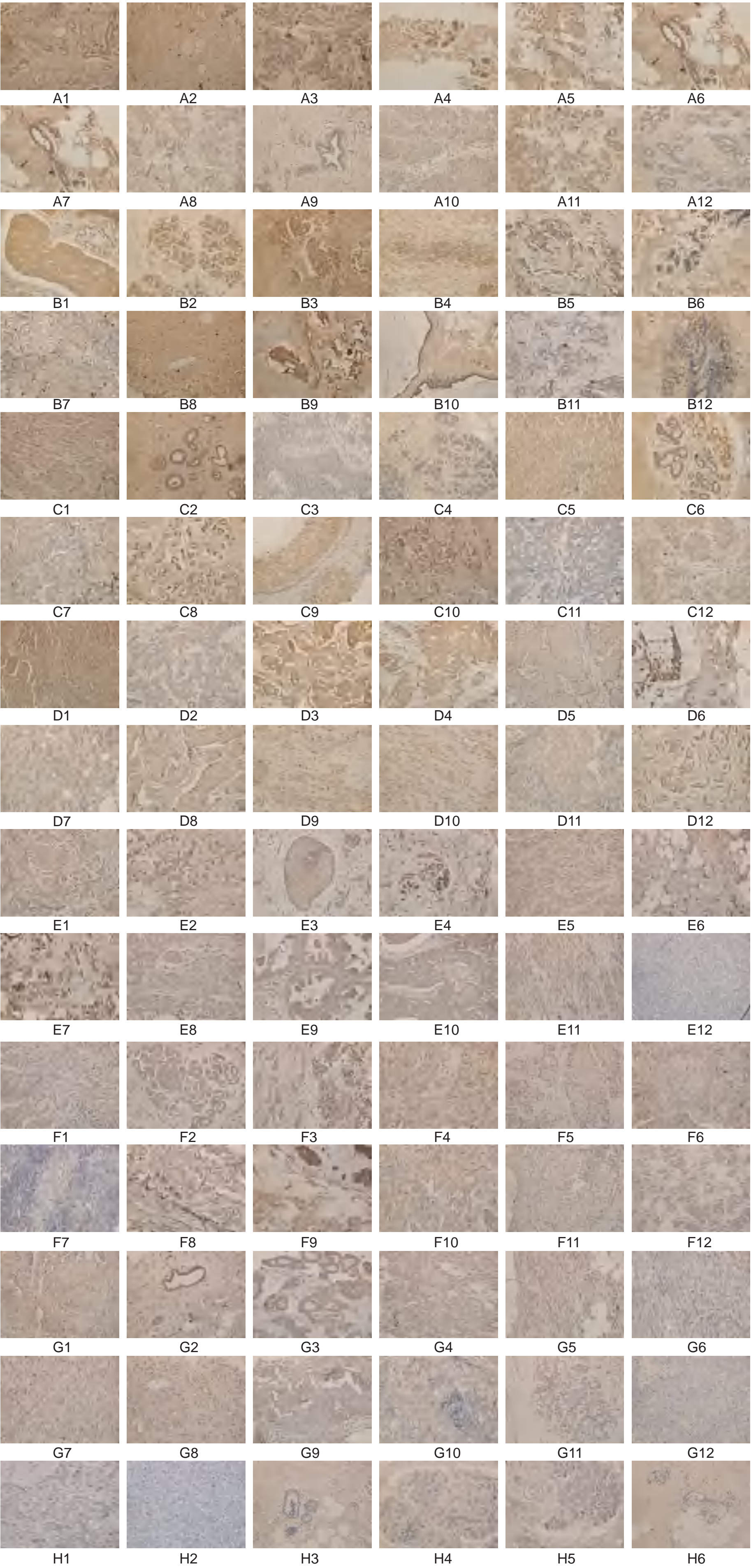

Supplement: Supplementary file 4 [file DataSheet1.PDF]
